# Misinformation About COVID-19 Vaccines on Social Media: Rapid Review

**DOI:** 10.2196/37367

**Published:** 2022-08-04

**Authors:** Ingjerd Skafle, Anders Nordahl-Hansen, Daniel S Quintana, Rolf Wynn, Elia Gabarron

**Affiliations:** 1 Faculty of Health, Welfare, and Organisation Østfold University College Halden Norway; 2 Faculty of Medicine University of Oslo Oslo Norway; 3 Department of Education, ICT, and Learning Østfold University College Halden Norway; 4 Department of Psychology University of Oslo Oslo Norway; 5 KG Jebsen Centre for Neurodevelopmental Disorders University of Oslo Oslo Norway; 6 Norwegian Centre for Mental Disorders Research (NORMENT) University of Oslo Oslo Norway; 7 NevSom Department of Rare Disorders & Disabilities Oslo University Hospital Oslo Norway; 8 Department of Clinical Medicine The Artic University of Norway Tromsø Norway; 9 Division of Mental Health and Substance Use University Hospital of North Norway Tromsø Norway; 10 Norwegian Centre for E-health Research University Hospital of North Norway Tromsø Norway

**Keywords:** social media, misinformation, COVID-19 vaccines, vaccination hesitancy, autism spectrum disorder

## Abstract

**Background:**

The development of COVID-19 vaccines has been crucial in fighting the pandemic. However, misinformation about the COVID-19 pandemic and vaccines is spread on social media platforms at a rate that has made the World Health Organization coin the phrase *infodemic*. False claims about adverse vaccine side effects, such as vaccines being the cause of autism, were already considered a threat to global health before the outbreak of COVID-19.

**Objective:**

We aimed to synthesize the existing research on misinformation about COVID-19 vaccines spread on social media platforms and its effects. The secondary aim was to gain insight and gather knowledge about whether misinformation about autism and COVID-19 vaccines is being spread on social media platforms.

**Methods:**

We performed a literature search on September 9, 2021, and searched PubMed, PsycINFO, ERIC, EMBASE, Cochrane Library, and the Cochrane COVID-19 Study Register. We included publications in peer-reviewed journals that fulfilled the following criteria: original empirical studies, studies that assessed social media and misinformation, and studies about COVID-19 vaccines. Thematic analysis was used to identify the patterns (themes) of misinformation. Narrative qualitative synthesis was undertaken with the guidance of the PRISMA (Preferred Reporting Items for Systematic Reviews and Meta-Analyses) 2020 Statement and the Synthesis Without Meta-analysis reporting guideline. The risk of bias was assessed using the Joanna Briggs Institute Critical Appraisal tool. Ratings of the certainty of evidence were based on recommendations from the Grading of Recommendations Assessment, Development and Evaluation Working Group.

**Results:**

The search yielded 757 records, with 45 articles selected for this review. We identified 3 main themes of misinformation: medical misinformation, vaccine development, and conspiracies. Twitter was the most studied social media platform, followed by Facebook, YouTube, and Instagram. A vast majority of studies were from industrialized Western countries. We identified 19 studies in which the effect of social media misinformation on vaccine hesitancy was measured or discussed. These studies implied that the misinformation spread on social media had a negative effect on vaccine hesitancy and uptake. Only 1 study contained misinformation about autism as a side effect of COVID-19 vaccines.

**Conclusions:**

To prevent these misconceptions from taking hold, health authorities should openly address and discuss these false claims with both cultural and religious awareness in mind. Our review showed that there is a need to examine the effect of social media misinformation on vaccine hesitancy with a more robust experimental design. Furthermore, this review also demonstrated that more studies are needed from the Global South and on social media platforms other than the major platforms such as Twitter and Facebook.

**Trial Registration:**

PROSPERO International Prospective Register of Systematic Reviews CRD42021277524; https://www.crd.york.ac.uk/prospero/display_record.php?ID=CRD42021277524

**International Registered Report Identifier (IRRID):**

RR2-10.31219/osf.io/tyevj

## Introduction

### Background

An unprecedented global effort has been undertaken to develop vaccines that protect against COVID-19. However, there is a grave concern that vaccine hesitancy will be a major obstacle to reaching herd immunity. In 2019, the World Health Organization (WHO) had already named vaccine hesitancy as 1 of 10 threats to global health [1]. Global vaccine distribution equity is also a major challenge. Figures from February 2022 show that 61.9% of the world’s population has received at least one dose of a COVID-19 vaccine, but only 10.6% of people in the Global South have received a dose [2]. Furthermore, the rate of people receiving a COVID-19 vaccine in some high-income countries where vaccines are available and free has dropped [3]. The WHO reiterates that COVID-19 vaccines remain critical and are considered effective against severe disease and death [4].

The reasons behind COVID-19 vaccine hesitancy are complex. Fear of side effects and concerns about the pace at which the vaccines were developed have been cited as primary reasons behind this hesitancy [5]. In addition, misinformation about COVID-19 and vaccines has spread on social media platforms at a rate that has made the WHO coin the phrase infodemic [6]. An infodemic is “too much information including false or misleading information in digital and physical environments during a disease outbreak” [7,8].

A well-known false claim is that the measles, mumps, and rubella (MMR) vaccine can cause autism [9]. The claim has since been empirically refuted many times but is still stated as a major concern for some parents [10]. Motta and Steccula [11] examined American public opinion data on MMR safety collected before and after a retracted 1998 study linking autism to MMR. The researchers detected a statistically significant increase in public concern about MMR safety following the retracted study and the media attention it received. This suggests that misleading vaccine information can impact public confidence in vaccines and cause skepticism about vaccines in general. Since the retracted 1998 study, groups of vaccine deniers or *antivaxxers* have grown, and claims that vaccines are harmful have spread to almost all vaccines [12]. Pullan and Dey [13] analyzed search patterns in Google Trends during the early stage of the pandemic in 2020 and found that search interest in COVID-19 vaccines had understandably increased, but also found that well-known antivaccine searches such as “autism” and “mercury” also had a growing presence and similar spikes as search patterns for COVID-19 vaccines. These results confirm that the false claim of associations between autism and MMR vaccines has become an argument for all types of vaccines and also possibly a concern when it comes to COVID-19 vaccines. Therefore, we examined whether misinformation on social media in recent times linked autism to COVID-19 vaccines.

Furthermore, vaccine hesitancy based on misinformation seems to be a worldwide phenomenon regardless of the uneven distribution of COVID-19 vaccines [4]. Social media plays a crucial role in disseminating both correct information and misinformation about infectious diseases and vaccines [14]. Wilson and Wiysonge [15] showed, in a global cross-national analysis of geographically coded tweets and vaccination rates from 166 countries, that there was a significant relationship between social media use and vaccine hesitancy. However, there has been a joint effort by several of the largest social media platforms and technology companies to combat the spread of misinformation about COVID-19 [16].

### Objective

We aimed to synthesize the existing research on misinformation about COVID-19 vaccines spread on social media platforms and its effects. The secondary aim was to gain insight and gather knowledge about whether misinformation about autism and COVID-19 vaccines is being spread on social media platforms. The following questions guided our inquiry: What is known about misinformation regarding COVID-19 vaccines spread on social media platforms? What is known about the effects of misinformation about COVID-19 vaccines spread on social media platforms? What is known about social media misinformation on COVID-19 vaccines concerning autism spectrum disorder?

## Methods

### Design

We followed the guidance from Cochrane Rapid Reviews [17]. We chose a rapid review protocol in line with the recommendations by Cochrane; that is, the need “for timely evidence for decision-making purposes including to address urgent and emergent health issues and questions deemed to be of high priority” [17]. The need to address vaccine hesitancy toward COVID-19 vaccines is an emergent health issue. The narrative qualitative synthesis was undertaken with the guidance of the PRISMA (Preferred Reporting Items for Systematic Reviews and Meta-Analyses) 2020 Statement [18] and the Synthesis Without Meta-analysis reporting guideline [19].

### Search Strategy and Selection Criteria

With the help and expertise of an information retrieval specialist, we used the following search string in this rapid review: (“misinformation” OR “disinformation” OR “information”) AND (“social media” OR “Facebook” OR “Twitter” OR “Instagram” OR “WhatsApp” OR “Telegram” OR “Tumblr” OR “Pinterest” OR “YouTube” OR “VKontakte” OR “Snapchat” OR “TikTok” OR “Weibo” OR “WeChat” OR “Reddit”) AND (“covid*” OR “corona*” OR “pandemic” OR “Sars-CoV-2” AND “vaccine*” OR “vaccination*”).

No date or language limitations were used. The full search strategy of the information retrieval specialist is available in Multimedia Appendix 1.

Publications were excluded if the studies were not original empirical research, if studies examined vaccines in general and not COVID-19 vaccines, if studies did not examine social media misinformation, and if data were gathered before the COVID-19 vaccine Pfizer-BioNTech phase 3 clinical trial [20].

### Data Collection Process and Extraction

This review was registered with the PROSPERO international register of systematic reviews (CRD42021277524). Systematic searches in the PubMed, PsycINFO, ERIC, EMBASE, Cochrane Library, and Cochrane COVID-19 Study Register databases were conducted by an information retrieval specialist on September 9, 2021. Duplicates were identified and removed by IS and EG. We used Rayyan [21] as the screening tool. Rayyan is a web application and mobile app for systematic reviews. It eases the process of the initial screening of abstracts and titles and helps researchers save time when they share and compare include-exclude decisions. All titles and abstracts were screened by IS and ANH independently. In the initial search, no date restriction was set. However, during the piloting of the title and abstract screening, IS and ANH discussed the fact that there were studies that explored misinformation about COVID-19 vaccines at a very early stage in the pandemic, before any COVID-19 vaccines were a reality. We decided that we needed a threshold date as to when we believed we found misinformation about the actual COVID-19 vaccines to be relevant, as misinformation at a very early stage would be about a potential vaccine. Therefore, we decided to include studies that were conducted during and after the Pfizer-BioNTech phase 3 clinical trial, because then the news about an actual vaccine was starting to spread around the world and thus starting to become a reality. We chose the Pfizer-BioNTech vaccine because it was the first COVID-19 vaccine to be approved by the WHO [22].

Of the 319 titles and abstracts screened, IS and ANH disagreed on 35. The disagreements were resolved through discussions between the 2 reviewers and if an extra opinion was needed, EG was consulted. Of these articles, 1 article was in German, 2 were in Spanish, and the rest were in English. IS can understand German and EG speaks Spanish. IS and EG performed a further assessment of the eligibility of the full-text records and conducted a pilot exercise using the same 10 full-text articles to calibrate and test the review form. After the screening, both reviewers assessed the articles that the other had excluded. ANH assisted with conflicts and discussed doubts surrounding the included or excluded articles. The data extraction from the included articles involved 2 reviewers (IS and EG), where IS extracted data using a piloted form and EG checked for the correctness and completeness of the extracted data. Data from the included articles were extracted based on design and study population, type of misinformation, effect of misinformation, misinformation about autism, ethical considerations, and social media channels. The agreed evidence was then synthesized narratively.

To synthesize the knowledge gathered about the types of misinformation, a thematic analysis was performed [23]. After the data extraction, IS gathered the data on the content of the misinformation. The data extracts on misinformation were then coded by ANH. IS and ANH searched for themes based on the codes and agreed upon 3 final themes of misinformation: conspiracies, medical misinformation, and vaccine development. EG approved the themes. Multimedia Appendix 2 provides an overview of the thematic analysis that was undertaken.

### Assessment of Risk of Bias

The risk of bias was graded according to the Joanna Briggs Institute (JBI) Critical Appraisal tool “Checklist for Analytical Cross-sectional Studies” [24] by 1 experienced reviewer (DSQ). The evaluation was based on answers to 8 questions (yes, no, or not applicable). The studies were classified as having low (>70%), moderate (40%-70%), or high (<40%) risk of bias. A complete overview of the assessment can be found in Multimedia Appendix 3 [25-69].

### Assessment of the Quality of the Evidence

One experienced reviewer (RW) assigned certainty of evidence ratings based on recommendations by the Grading of Recommendations Assessment, Development and Evaluation (GRADE) working group [70]. The included studies that looked at associations were given a narrative GRADE score related to the outcome “Association between social media misinformation and vaccine hesitancy.” The level of quality of evidence was classified as very low, low, moderate, or high. A complete overview of the assessment can be found in Multimedia Appendix 4 [25-69].

### Data Synthesis

Narrative synthesis was undertaken with the guidance of the PRISMA 2020 Statement [18] and Synthesis Without Meta-analysis reporting guideline [19]. In the synthesis, findings from our included studies were grouped according to study design, population, social media sample, types of social media, types of misinformation reported, misinformation about autism, the reported effect of the misinformation on vaccine hesitancy, and the assessments of risk of bias and quality of evidence. When synthesizing the findings narratively, studies with a low risk of bias or high quality of evidence will be highlighted on several occasions.

## Results

### Study Selection and Risk of Bias

**Figure 1:**
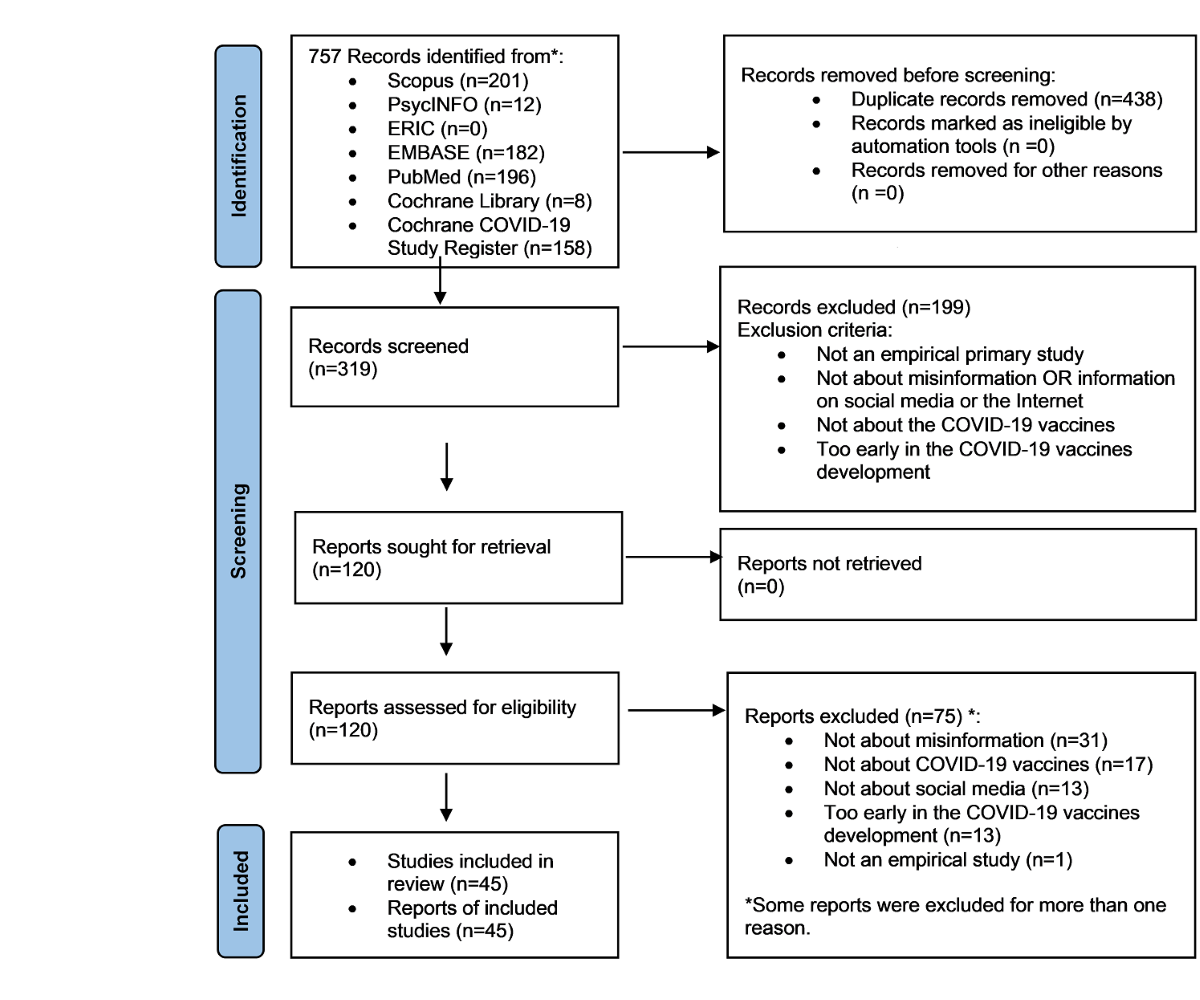
PRISMA (Preferred Reporting Items for Systematic Reviews and Meta-Analyses) 2020 flow diagram for new systematic reviews.

We identified 45 relevant studies (Figure 1). The list of excluded articles during the full-text review and the reasons for exclusion are reported in Multimedia Appendix 5. The risk of bias in 53% (24/45) of the included studies was classified as low, according to the JBI Critical Appraisal tool [25-42,47-52]. In total, 18% (8/45) of the studies showed a moderate risk of bias [43-46,53-56]. Finally, 27% (12/45) of the included studies showed a high risk of bias [57-68]. Of the 45 studies, in 1 (2%) study [69], none of the questions in the JBI tool were applicable.

We grouped the studies into 2 major categories according to data sampling. One group gathered data through surveys, interviews, or focus groups (Table 1). The other group gathered data from social media platforms (Table 2). The largest total population sample in the first group of 22 studies (Table 1) came from Europe, with 27,975 respondents in total. All respondents were described as adults or >18 years, except for 2 studies in the United States where the participants were aged ≥65 years [28,34]. Another exception was 1 study from Slovenia, where participants aged ≥15 years were included [35]. Gender has not been a focal point in any of the 45 included studies.

Data were extracted from social media platforms in 23 of the included studies. These studies formed the second group (Table 2).

The 12 studies that were assessed to have a high risk of bias were found in the second group (Table 2), whereas the studies in Table 1 had a low or moderate risk of bias according to the JBI tool.

**Figure 2:**
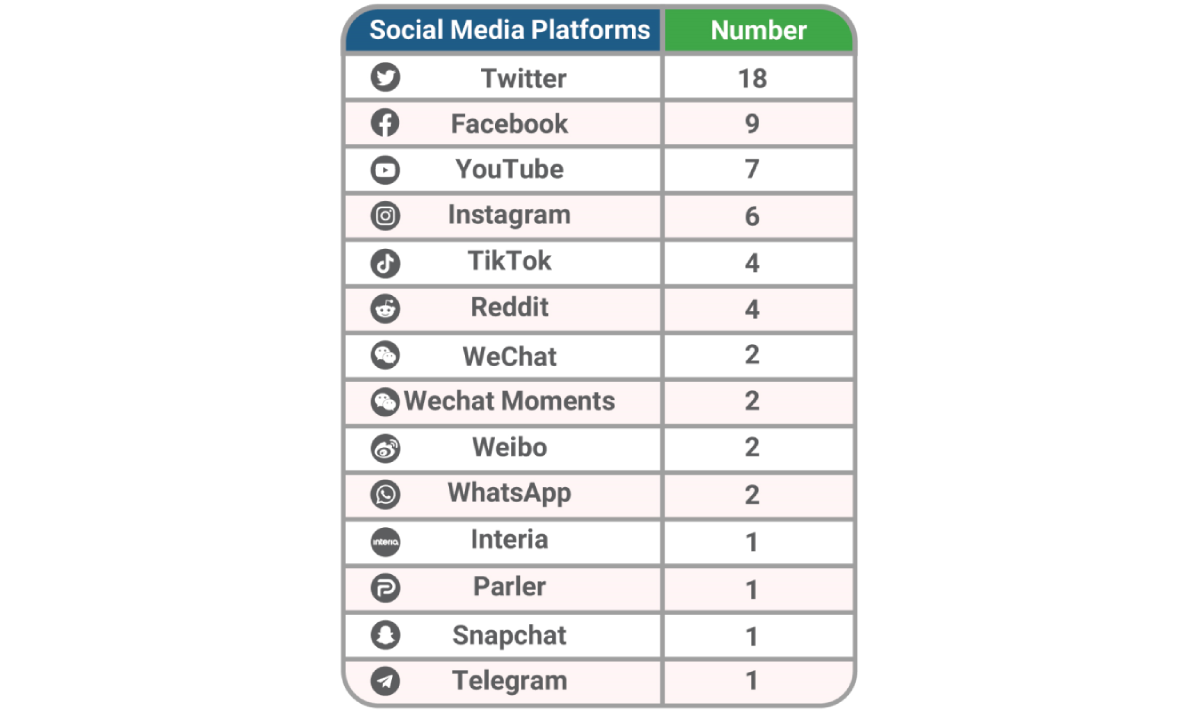
Social media platforms.

Many of the studies did not name social media platforms in the first group (Table 1) but rather discussed social media platforms in general. However, some studies did specify which social media platforms they were assessing. Figure 2 summarizes the types of social media platforms specified in the 45 included studies.

**Table 1:** Studies in which data were collected through surveys, observations, or interviews (n=22).

Study	Country	Study period	Study design	Type of social media	Social media or population sample	Type of misinformation reported	Risk of bias (JBI^a^)
Alibrahim and Awad [25], 2021	Kuwait	March 26 to April 26, 2021	Cross-sectional study	Not specified	4147 adults, ≥18 years	COVID-19 is not a serious infection that requires vaccination	Low
Allington et al [26], 2021	United Kingdom	November 21 to December 21, 2020	Cross-sectional study	Not specified	4343 UK residents, aged 18-75 years	Conspiracy theories (not specified)	Low
Aloweidi et al [27], 2021	Jordan	January 22 to February 28, 2021	Cross-sectional study	Not specified	646 adults	The vaccines are unsafe; effect of the vaccines on a genetic level; causes chronic illnesses; may lead to infertility; can affect their offspring; contains toxic heavy metals and neurotoxic materials; it is a part of a secret research	Low
Bhagianadh and Arora [28], 2021	United States	October to November 2020	Longitudinal survey	Not specified	5784 Medicare enrollees, ≥65 years	Distrust of government narrative about vaccines; vaccine will cause COVID-19	Low
Brodziak et al [29], 2021	Poland	January 26 to February 28, 2021	Survey	Not specified	635 adult patients with cancer	The vaccine contains bodies of aborted children; COVID-19 does not exist	Low
Chadwick et al [30], 2021	United Kingdom	September 24 to October 17, 2020	Cross-sectional study	Not specified	5114 adults in the United Kingdom	Conspiracies (not specified)	Low
Ebrahimi et al [31], 2021	Norway	January 23 to February 2, 2021	Cross-sectional study	Not specified	4571 Norwegian adults	Not specified	Low
Kanyike et al [32], 2021	Uganda	Monday, March 15, and Sunday, March 21, 2021	Cross-sectional study	Not specified	600 medical students, ≥18 years	Negative information about COVID-19	Low
Karabela et al [33], 2021	Turkey	February 1, 2021, to February 28, 2021	Cross-sectional study	Social media, WhatsApp, and YouTube	1216 adults	Conspiracy theories (not specified)	Low
Park et al [34], 2021	United States	October to November 2020	Cross-sectional study	Not specified	6478 Medicare beneficiaries	The belief that COVID-19 is not that dangerous	Low
Petravić et al [35], 2021	Slovenia	December 17 to December 27, 2020	Cross-sectional study	Not specified	12,042 Slovenian residents, ≥15 years. Analysis of responses from the 2320 respondents (12%) who answered the open-ended question	The vaccines will cause a genocide; COVID-19 is the same as influenza	Low
Sallam et al [36], 2021	Jordan	January 19 to January 23, 2021	Cross-sectional study	Not specified	1106 university students	COVID-19 was man-made for enforcing vaccinations; COVID-19 vaccinations intends to implant microchips into people to control them; COVID-19 vaccination will lead to infertility	Low
Sallam et al [37], 2021	Jordan, Kuwait, and Saudi Arabia	December 4 to December 18, 2020	Cross-sectional study	Facebook, Instagram, Twitter, and WhatsApp	3414 respondents	An artificial origin of the virus; the disease was man-made to enforce vaccination; microchip implanting and infertility claims	Low
Sharevski and Gover [38], 2021	United States	January and February 2021	Cross-sectional quasi-experimental study	Twitter	304 respondents, ≥18 years	Exaggeration of rare side effects of COVID-19 vaccines	Low
Zhang et al [39], 2021	China	September 1 to September 7, 2020	Cross-sectional study	WeChat, WeChat moments, Weibo, TikTok	2053 Chinese factory workers (full-time employees) ≥18 years	Negative information about COVID-19 vaccines	Low
Zhang et al [40], 2021	China	September 1 to September 7, 2020	Cross-sectional study	WeChat, WeChat moments, Weibo, TikTok	2053 Chinese parents, ≥18 years	Negative information about COVID-19 vaccines	Low
Costantino et al [41], 2021	Italy	December 2020 to March 2021	Cross-sectional study	Not specified	363 adults	Unfavorable information about COVID-19 vaccines	Low
Jennings et al [42], 2021	United Kingdom	Survey: December 12 to December 18, 2020. Focus groups: November 30 to December 7, 2020	Cross-sectional qualitative and quantitative (mixed method) study	TikTok, Instagram, Snapchat, Twitter; Facebook, YouTube	1476 UK adults participated in the survey; 29 adults in the United Kingdom participated in the focus groups	Conspiracy theories (not specified)	Low
El-Far Cardo et al [43], 2021	Germany	August and November 2020	Cross-sectional study	Facebook, Twitter, Telegram	808 persons	COVID-19 is not a health threat	Moderate
Knights et al [44], 2021	United Kingdom	June 18 and November 30, 2020	Cross-sectional qualitative study	Not specified	64 primary care professionals and administrative staff and 17 recently arrived migrants	5G conspiracy theory	Moderate
Berry et al [45], 2021	United States	December 30, 2020, to January 15, 2021	Qualitative observational study	Not specified	193 skilled nursing facility workers	Vaccines cause COVID-19; microchip; the virus has been around for a long time and killed many people since 1918; fear of racist motives and the safety of the vaccines; the vaccines have fetal cells from abortions	Moderate
Choudhary et al [46], 2021	India	February 18 to February 28, 2021	Cross-sectional study	Not specified	272 Indian adults, ≥18 years	COVID-19 is a conspiracy	Moderate

^a^JBI: Joanna Briggs Institute.

**Table 2:** Studies in which data were collected from social media platforms (n=23).

Study	Country	Study period	Study design	Type of social media	Social Media or population sample	Type of misinformation reported	Risk of bias (JBI^a^)
Chan et al [47], 2021	The United Kingdom	December 10, 2020	Cross-sectional study (social media data extraction)	YouTube	48 COVID-19 vaccine–related videos on YouTube	Misinformation about COVID-19 vaccines (not specified). Only 2 (4.2%) videos made nonfactual claims.	Low
Herrera-Peco et al [48], 2021	Spain	December 14 to December 28, 2020	Cross-sectional study (social media data extraction)	Twitter	5040 Twitter users participated, generating a total of 1,664,261 impressions	Messenger RNA vaccines will produce changes in human DNA; government and pharmaceutical industries are allies; adverse effects leading to genocide.	Low
Hughes et al [49], 2021	United States	October 2020 to November 2020	Cross-sectional study (social media data extraction and modeling)	Facebook, Twitter, YouTube, and Instagram	Using hashtag and keyword searchers, a team of subject matter experts identified 20 channels (ie, bounded sources of content, such as a social media account), which appeared to contain a high degree of antivaccine content or COVID denialism.	Corrupt elites; physical deformities; mental illness; microchips that violate your autonomy and privacy; the people who intentionally created the COVID vaccine are shadowy and suspicious.	Low
Larrondo-Ureta [50], 2021	Spanish-speaking countries	December 2020 and February 2021	Cross-sectional study (social media data extraction)	Twitter	62,045 tweets and 258,843 retweets	Antivaccine discourse (not specified)	Low
Liu and Liu [51], 2021	English-speaking countries	November 1 to November 22, 2020	Cross-sectional study (social media data extraction)	Twitter	5000 COVID-19 vaccine–related tweets, which were posted by 4796 unique users.	Microchips; alters DNA; women become sterile.	Low
Sobkowicz and Sobkowicz [52], 2021	United States and Poland	March 1, 2021	Cross-sectional study (social media data extraction)	Reddit and Interia	Reddit and Interia antivaccine groups	Antivaccination discussions about COVID-19 vaccines.	Low
Guntuku et al [53], 2021	United States	December 1, 2020, to February 28, 2021	Cross-sectional study (social media data extraction)	Twitter	78.1 million vaccine-related tweets	Evangelical hubs posted conspiracy theories about Bill Gates and China.	Moderate
Hernández-García et al [54], 2021	Spain	February 9 2021	Cross-sectional study (social media data extraction)	YouTube	118 YouTube videos	Hoaxes and conspiracy theories (not specified).	Moderate
Islam et al [55], 2021	Australia	December 31, 2019, to November 30, 2020	Cross-sectional study (social media data extraction)	Facebook, YouTube, and Twitter	637 news articles, social media narratives, web-based reports, and blogs spread on social media	Daughter of the Russian president had died after receiving the second dose of COVID-19 vaccine; children and soldiers dying after receiving the vaccine in multiple countries; conspiracy theory about Bill Gates; COVID-19 vaccine can monitor the human population and take over the world; COVID-19 vaccines contain a microchip through which biometric data could be collected, and large businesses could send signals to the chips using 5G networks; crucial phases of the clinical trials were skipped; COVID-19 vaccine contains cells from aborted fetus or genes from pigs.	Moderate
Kwok et al [56], 2021	Australia	January 22 and October 20, 2020	Cross-sectional study (social media data extraction)	Twitter	31,100 COVID-19 vaccine–related tweets	Conspiracy theories such as the “mark of the beast” and microchips in vaccines.	Moderate
Alliheibi et al [5,8], 2021	Saudi Arabia	December 15, 2020, to May 25, 2021	Cross-sectional study (social media data extraction)	Twitter	37,467 Arabic tweets from 23,748 users	COVID-19 vaccination is a cover for a plan devised by Bill Gates to implant trackable microchips to control people.	High
Baines et al [58], 2021	United States	November 20, 2020, to January 6, 2021	Cross-sectional study (social media data extraction)	Parler	400 random parleys from a large sample of 7000 parleys	Sterilization possibilities for men and women; COVID-19 vaccine to control the population; Bill Gates and Anthony Fauci had instigated measures (ie, microchips and enzymes in the vaccine) to control the population through the administration of the COVID-19 vaccine; governments and certain powerful individuals “planned” this health crisis to vaccinate children without parental consent as part of the new world order to control future populations.	High
Basch et al [59], 2021	United States	December 2020	Cross-sectional study (social media data extraction)	TikTok	100 videos studied garnered 35,338,600 views	38 videos discouraged the vaccine; 3 videos claimed that the vaccine is a hoax.	High
Boucher et al [60], 2021	Canada	November 19 and November 26, 2020	Cross-sectional study (social media data extraction)	Twitter	636,516 English and French tweets	COVID-19 vaccines are poison and the messenger RNA technology has not been tested yet and is harmful.	High
Criss et al [61], 2021	United States	October 2020 to January 2021	Cross-sectional study (social media data extraction)	Twitter	1110 tweets	Misleading information that countered scientific research about the vaccines; the government using vaccines to insert microchips and control the population; the immune system is stronger than the vaccines; race extermination conspiracy that claims that the vaccine was created to “kill off [people of color] POC.”	High
Herrera-Peco et al [62], 2021	Spain	December 8 to December 23, 2020	Cross-sectional study (social media data extraction)	Twitter	6080 Twitter interactions (n=499 of those are single tweets)	Deny the existence of the virus; the vaccine will modify the DNA of human beings; industry lobbies to kill older adults and leave young adults with Bells syndrome.	High
Melton et al [63], 2021	United States	December 1, 2020, to May 15, 2021	Cross-sectional study (social media data extraction)	Reddit	13 Reddit communities	Misinformation about side effects.	High
Pascual-Ferrá et al [64], 2021	United States	December 29, 2019, to January 2, 2021	Cross-sectional study (social media data extraction)	Facebook, Instagram, Reddit, and YouTube	Peaks and interactions	Viral video of a nurse fainting after vaccine uptake. Misinformation about COVID-19 vaccines (not specified).	High
Rotolo et al [65], 2021	United States	March 19, 2020, and June 16, 2021	Cross-sectional study (social media data extraction)	Facebook, Twitter, and Instagram	Aim: share 49 infographics to counter vaccine hesitancy.	COVID-19 myths.	High
Savolainen [66], 2021	Finland	February 2021	Cross-sectional study (social media data extraction)	Reddit, from the subreddit VaxxHappened	40 threads contained in total 1877 messages	Misinformation about COVID-19 vaccines (not specified).	High
Thelwall et al [67], 2021	United Kingdom	March 10 to December 5, 2020	Cross-sectional study (social media data extraction)	Twitter	446 COVID-19 vaccine–hesitant tweets in English	Deep state conspiracy; depopulation; microchips; Bill Gates; fearing that people of color are at risk for experimentation—motivated by the infamous US federal government Tuskegee Syphilis study ending in 1972 that secretly experimented on poor African American men.	High
Wawrzuta et al [68], 2021	Poland	November 1, 2020, to May 1, 2021	Cross-sectional study (social media data extraction)	Facebook	3414 Facebook comments	The vaccine was created only for the profit of pharmaceutical companies; conspiracy theories, hidden vaccine effects (eg, chips); the vaccine will be dangerous to health; the vaccine has existed before the COVID-19 pandemic.	High
Doyno et al [69], 2021	Unites States	January to April 2021	Quasi-experimental study	YouTube, Twitter, Facebook, and Instagram	Information campaign with 79 COVID-19 vaccine–related videos in English, Cantonese, Spanish, Mandarin, and Polish	Misinformation (not specified).	N/A^b^

^a^JBI: Joanna Briggs Institute.

^b^N/A: not applicable.

### Thematic Analysis

**Figure 3:**
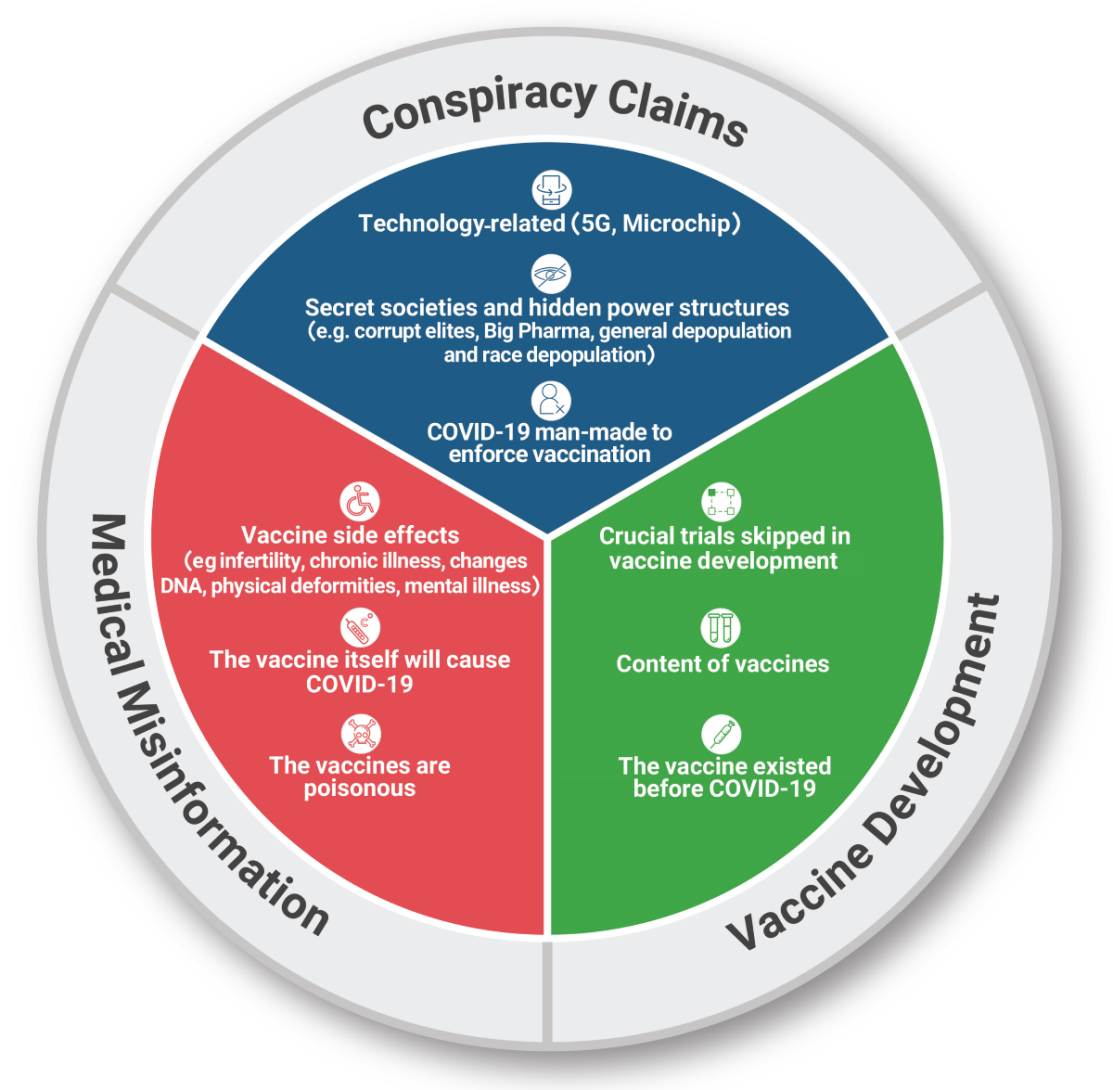
Types of misinformation about the COVID-19 vaccine on social media platforms.

Figure 3 provides an overview of the 3 overarching themes of misinformation identified from the thematic analysis, and examples from the data extraction and codes that laid ground for the final 3 themes are seen in the inner circles of the figure.

Of the 45 included studies, 18 (40%) studies reported misinformation across all 3 categories [27-29,35-37, 45,48,49,51,55,57, 58,60-62,67,68], 9 (20%) studies reported only on conspiracies [26,30,33,42,44,46,53,54,56], 6 (13%) studies were concerned specifically with medical misinformation [25,34,38,43,63,64], and 12 (27%) studies reported on COVID-19 vaccine misinformation or antivaccine discourse without going into further detail [31,35,39-41,47,52,59,65,66,69].

### Effects of Social Media Misinformation

We identified 19 studies that made assumptions regarding the effects of social media misinformation on vaccine hesitancy (Table 3). The evaluation of the certainty of evidence of these 19 studies that measured the “Association between social media misinformation and COVID-19 vaccine hesitancy” was classified as moderate or low to moderate according to GRADE in 2 cases [38,65]. For the rest of the studies, the certainty of evidence according to GRADE was considered low or very low.

The 2 studies with a higher certainty of evidence had an experimental design. Rotolo et al [65] aimed to develop and distribute infographics that addressed COVID-19 vaccine hesitancy and misinformation. Although their infographics reached thousands of people, they were unable to determine the impact on vaccine hesitancy. Sharevski and Gover [38] analyzed the perceived accuracy of COVID-19 vaccine–related tweets when they were moderated by smart device technology that Twitter applies to COVID-19 misinformation. The results from the 304 participants suggested that vaccine-hesitant users ignored warnings as long as the tweets aligned with their personal beliefs.

**Table 3:** Studies in which the effect of social media misinformation is measured or discussed (n=19).

Study	Reported effect of misinformation	Certainty of evidence (GRADE^a^)
Sharevski and Gover [38], 2021	Amazon Alexa was not able to dispel any biases that were rooted in personal beliefs. One’s hesitancy from COVID-19 vaccination sufficed for biased perception of the information from Alexa despite any labeling as long as the tweets echoed their skeptical outlook on the whole COVID-19 vaccination effort.	Moderate
Rotolo et al [65], 2021	Each infographic reached thousands to tens of thousands of people. We do not know whether those who viewed these infographics changed their perspective on vaccination, so we are unable to conclude their impact on vaccine hesitancy based on this study alone.	Low to moderate
Allington et al [26], 2021	Informational reliance on all social media platforms was positively correlated with vaccine hesitancy; this correlation was strongest concerning Facebook and YouTube (R_S_^b^=0.15 and R_S_=0.18, respectively). Coronavirus conspiracy suspicions and general vaccine attitudes appear uniquely predictive, jointly explaining 35% of variance.	Low
Bhagianadh and Arora [28], 2021	Those depending on social media as the main source of information on COVID-19 expressed higher negative vaccine intent (OR^c^ 3.36, 95% CI 1.44-7.82). Among those who expressed a negative vaccine intent, 40% (n=298) expressed no trust in government, and 10% (n=74) said that the vaccines cause COVID-19.	Low
Boucher et al [60], 2021	The study showed 2 clusters opposite to these vaccine acceptant clusters exhibiting more vaccine-hesitant narratives. There were 23.4% (n=146,191) of conversations on Twitter during this period of observation that can be directly attributed to vaccine hesitancy.	Low
Chadwick et al [30], 2021	Combinations of news avoidance and high levels of the news-finds-me attitude and social media dependence and high levels of conspiracy mentality are most likely to be associated with web-based discouragement of vaccination.	Low
Jennings et al [42], 2021	Holding conspiracy beliefs is a significant predictor of vaccine hesitancy. In the bivariate analysis, there is some support for a relationship between social media use (Snapchat, TikTok, YouTube, and Instagram) and increased vaccine hesitancy. YouTube users were significantly less willing to be vaccinated, with a two-thirds likelihood of vaccine willingness compared with nonusers.	Low
Liu and Liu [51], 2021	279 tweets stated their behavioral intentions. A total of 97 tweets were labeled with positive behavioral intentions, while 182 tweets contained negative behavioral intentions.	Low
Park et al [34], 2021	The study found that social media dependence and high levels of conspiracy mentality were most likely to be associated with web-based discouragement of vaccination. The likelihood of COVID-19 vaccine uptake was significantly lower among those relying on social media (OR 0.40, 95% CI 0.25-0.65)	Low
Zhang et al [39], 2021	Regarding social media influence, higher frequency of exposure to positive information related to COVID-19 vaccination was associated with a higher intention to receive a COVID-19 vaccination at market rate (AOR^d^ 1.53, 95% CI 1.39-1.70) or a free vaccination (AOR 1.52, 95% CI 1.35-1.71).	Low
Zhang et al [40], 2021	Higher exposure to positive information related to COVID-19 vaccination on social media was associated with higher parental acceptability of COVID-19 vaccination (AOR 1.35, 95% CI 1.17-1.56). Higher exposure to negative information related to COVID-19 vaccination was negatively associated with the dependent variable (AOR 0.85, 95% CI 0.74-0.99).	Low
Aloweidi et al [27], 2021	The effect of social media (OR 1.21, 95% CI 1.04-1.41; *P*=.01) was significantly associated with the willingness to take COVID-19 vaccine. Circulated information about COVID-19 vaccines on social media platforms that they believed in: it is unsafe (n=283, 43.8%); effect of the vaccines on a genetic level (n=87, 13.5%); causes chronic illnesses (n=60, 9.3%); may lead to infertility (n=43, 6.7%); can affect their offspring (n=56, 8.7%); toxic heavy metals and neurotoxic materials (n=47, 7.3%); it is a part of a secret research (n=101, 15.6%)	Very low to low
Brodziak et al [29], 2021	A total of 432 (68%) used social media every day. Unwilling to vaccinate against COVID-19: social media as a source of information about vaccinations (OR 1.42, 95% CI 0.72-2.80). Not a significant predictor; attitudes toward COVID-19 vaccines: afraid of the vaccine’s side effects (n=284, 44.7%); afraid of the composition of the vaccine (n=239, 37.6%); contains bodies of aborted children (n=49, 7.7%); COVID-19 does not exist (n=42, 6.6%)	Very low to low
Ebrahimi et al [31], 2021	Individuals with a preference for social media platforms as compared with those preferring source-verified media platforms had a near 2-fold (ie, 1.64) odds of being hesitant toward vaccination. Belief in superiority of natural immunity: OR 2.663, 95% CI 2.350-3.028; *P*<.001	Very low to low
El-Far Cardo et al [43], 2020	Factors that were negatively associated to get vaccinated were using social media in general as an information source about COVID-19 (*P*=.01) and the use of Facebook (*P*=.05) or Telegram (*P=*.05). However, using Twitter was not significantly associated with adverse vaccination intentions (*P=*.56). Believing that COVID-19 is not dangerous was associated with unwillingness to get vaccinated.	Very low to low
Petravić et al [35], 2021	Those who trusted alternative media sources (alternative explanations on social media) and had a distrust of the government were more vaccine hesitant.	Very low to low
Sallam et al [36], 2021	The lowest rate of intention to get the vaccine was among those who depended on social media platforms (19.8%) compared with dependence on medical doctors, scientists, and scientific journals (47.2%, *P*<.001). Conspiracy beliefs were evaluated using the validated VCBS^e^, with higher scores implying embrace of conspiracies. A significantly higher VCBS score was correlated with reluctance to get the vaccine (*P*<.001).	Very low to low
Costantino et al [41], 2014	A total of 71.4% (n=60) responded that unfavorable information about COVID-19 vaccines obtained from the internet, social media, or media was associated with the decision to not take the vaccine.	Very low
Karabela et al [33], 2021	Although the correlation was not significant, of the participants, those who considered having vaccination mostly trusted YouTube as their source of information. In contrast, the participants who stated that they would have the COVID-19 vaccine did not trust social media sites such as Facebook, Twitter, and Instagram (*P*<.005). There was a positive and low-level relationship between attitudes toward COVID-19 vaccines and conspiracy theories (*r*=0.214).	Very low

^a^GRADE: Grading of Recommendations Assessment, Development and Evaluation.

^b^R_S_: Spearman Rank Correlation Coefficient.

^c^OR: odds ratio.

^d^AOR: adjusted odds ratio.

^e^VCBS: Vaccine Conspiracy Belief Scale.

Allington et al [26] analyzed findings from a web-based survey conducted with a sample of 4343 adults in the United Kingdom. They found a positive correlation between trust in social media and vaccine hesitancy and the strongest link was found for YouTube and Facebook. Conspiracy suspicions about COVID-19 and general vaccine attitudes appeared to be uniquely predictive, jointly explaining 35% of the variance. Boucher et al [60] analyzed 636,516 English and French tweets. A total of 23.4% (n=146,191) of the conversations on Twitter during the study period could be directly attributed to vaccine hesitancy. A British study by Liu and Liu [30] of 5114 adults found that social media dependence and high levels of conspiracy mentality were most likely to be associated with web-based discouragement of vaccination. In a study of 4571 Norwegian adults, individuals who preferred social media platforms had nearly 2-fold (ie, 1.64) odds of being hesitant toward COVID-19 vaccination compared with those preferring source-verified media platforms [31]. In addition, those who held the belief of the superiority of natural immunity over vaccination were more vaccine hesitant (odds ratio 2.663, 95% CI 2.350-3.028; *P*<.001). Petravić et al [35] asked 12,042 Slovenian residents about their attitudes toward COVID-19 vaccines. Those who trusted alternative media sources and alternative explanations on social media were more vaccine hesitant. A total of 11 studies [27-29,33,34,36,39-43] discussed social media misinformation, vaccine uptake, and vaccine intentions.

## Discussion

### Principal Findings

The 45 included studies about misinformation on social media platforms about COVID-19 vaccines suggest that there should be great concern about the volume of misinformation being spread, and the association between COVID-19 vaccine misinformation and vaccine hesitancy. To our knowledge, this is the first review to analyze social media misinformation about COVID-19 vaccines. We identified 3 overall categories of misinformation, namely, medical misinformation, conspiracies, and distrust in vaccine development; however, the 3 categories are connected and sometimes overlapping, as distrust in vaccine development might be founded in conspiratorial beliefs about hidden power structures and corrupt elites. The included studies were predominantly from Europe and the United States, and therefore, there is a lack of information, especially from African and South American countries. Twitter was the most studied platform, with Facebook and YouTube being in the second and third place, respectively.

Fear of side effects is a major concern when it comes to vaccine hesitancy, and as this review shows, this concern can easily turn into medical misinformation and exaggerations of side effects. To synthesize what is known about social media misinformation about COVID-19 vaccines from the included studies, a thematic analysis was undertaken. The coded extract of data that made up the theme medical misinformation contained misinformation about side effects such as infertility, chronic illness, changes in DNA, physical deformities, and mental illness. Only one study mentioned autism as an adverse side effect of COVID-19 vaccines [63]. Knowing that the side effects of the vaccines are a major concern [5], medical misinformation has the potential to do a lot of harm.

When we examined the types of reported misinformation, we also found that a lot of misinformation is grounded in conspiracy theories. Some of these conspiracy theories have become infamous, such as the belief that there are secret societies and hidden power structures run by corrupt elites. These elites are believed to be networking with big pharmaceutical companies to make money or to depopulate the world. There are also conspiracy theories about racially motivated depopulation. For example, we found 3 studies from the United States that mentioned the fear of racist motives by official health authorities as a reason for vaccine hesitancy [45,61,67]. Some of this fear has historical roots in the United States, as one of these studies [67], for instance, brought up the Tuskegee Syphilis study. This was a clinical study (1932-1972) in which the United States Public Health Service used African Americans to observe untreated syphilis and therefore denied them treatment [71]. This exemplifies that a lack of trust in public health institutions might have deep historical roots in some countries and cultures. Other issues to be aware of are religious concerns and vaccine hesitancy. We found several studies that reported on misinformation about the content and development of vaccines and in some studies [29,45,55], we found very explicit language (eg, “pigs” and “cells from aborted children”). Such wording can cause worry in some religious communities.

The second objective of this review was to examine the effects of social media misinformation about COVID-19 vaccines. The 19 studies identified in Table 3 interpreted the results as associations among social media use, misinformation, and vaccine hesitancy. According to the JBI and GRADE evaluations, there is a need for more robust designs to become more certain regarding the actual effect of social media misinformation on vaccine hesitancy. Only 1 study, an intervention study regarding the impact of addressing misinformation on Twitter users, was assessed to have a low risk of bias and moderate quality of evidence [38]. In addition, 4 studies reported significance levels of associations, but the effect size was not reported [31,32,35,50]. Other studies in this review showed that social media platforms did not necessarily spread misinformation to a great extent, perhaps reflecting that the effort made by some social media platforms to halt misinformation has worked. Chan et al [47] examined 48 COVID-19 vaccine–related videos on YouTube in December 2020 and found only 2 videos (4.2%) that made nonfactual claims. Hernández-García et al [54] also examined YouTube videos during February 2021 and found that only 2 out of 110 videos contained COVID-19 vaccine hoaxes or conspiracy theories. Pascual-Ferrá et al [64] examined social media data from Facebook, Instagram, Reddit, and YouTube and did not find evidence of the dominance of misinformation. However, what is being spread and discussed in closed groups is another question that needs to be examined further. Another valid approach would also be to examine comment sections. Although antivaccine content has been prevented from surfacing in searches, this does not prevent people from commenting about their beliefs or posting other types of information in the comment section. It is controversial to deplatform people [72] and might even do harm, as these people might be seen as someone speaking against the establishment, which are, in essence, some of the core beliefs of some conspiracy theorists.

Surprisingly, there was a dearth of studies examining misinformation about autism and COVID-19 vaccines. Considering the history of misinformation about vaccines and autism over the past 2 decades, more research should focus on this topic. One could also speculate whether this would have played out differently if COVID-19 vaccines were more targeted toward younger children. Future research should also aim to examine social media platforms such as TikTok, which is a very popular platform worldwide, and is often used by people who are younger than, for instance, the average Twitter user [73]. The low inclusion of some social media platforms such as TikTok or Telegram is a limitation, as certain parts of the population and particular communities are not included.

When addressing vaccine hesitancy, one should be careful before labeling all vaccine-hesitant people as antivaxxers or misinformed people. The primary concerns from people who say that they are vaccine hesitant are the safety of the vaccines and the rapid pace of their development [7]. However, being hesitant and skeptical does not mean that these people are unwilling to take the vaccines but rather that they have some concerns that should be adequately addressed to convince them of the safety and efficacy of the vaccines. To understand a complex issue such as vaccine hesitancy, knowledge about sociodemographic conditions and cultural awareness is key. In addition, countries with a more undemocratic regime will suffer from a lack of trust in official authorities, which may damage an official vaccine campaign. People’s trust in the government varies between countries and cultures. Although some countries have a tradition for mandatory vaccination, this is less acceptable in other countries.

The issue of trust is also an important issue to be considered. “Fake news” became a buzzword in the last decade and the term was used not only to actually coin false news but also to spread distrust to news agencies and official actors, accusing them of spreading falsehoods. Vosoughi et al [74] aimed to understand how false news spread and examined a set of rumors (n=126,000) spread by 3 million people on Twitter from 2006 to 2017. The results showed that false news spread much faster and reached a larger audience than real news. Social media has contributed to a far more complex information landscape than before and has created new challenges when it comes to building trust in official actors. These are issues that need to be addressed and analyzed in future studies of misinformation about vaccines.

### Limitations

We did not include gray literature or preprints in this review. The rapid pace at which the pandemic is moving makes preprint research particularly relevant. However, although peer review is not a guarantee of quality, we decided not to include gray literature or preprints and limited eligible articles to peer-reviewed manuscripts. We did not contact researchers with potential projects on this subject matter. Furthermore, the searched databases were selected based on the topic at hand. There will always be a chance that other, more specified or general databases would capture other studies.

A limitation of the evidence included in this review is that, in our assessment, all but one received a low score on the assessment of quality of evidence. However, it is a challenge in the process of assessing quality of evidence and risk of bias, that the included studies have a range of different designs, each with its strengths and weaknesses. We applied 2 tools in this regard: 1 from the JBI [24] and 1 from the GRADE Working Group [70]. Neither tool provides a complete picture of the included studies, but they may help the reader in obtaining a broader view of the included studies.

Furthermore, there is a poor correlation between self-reported social media use and actual use [75]. A high proportion of these studies extracted data from Twitter because Twitter has opened up access for researchers to extract data from its platform, making it more accessible compared with other social media platforms. The Twitter sample may not be representative of a random sample of the population, as its users tend to range in age from 25 to 34 years and are predominantly from the United States [76,77]. Furthermore, we did not assess the potential presence of social media bots (automated accounts) spreading incorrect information in these studies. We also did not discuss how social media algorithms partake in creating echo chambers [78]. These are well-known challenges in researching data gathered from social media [79]. Only 1 study included in this review was from an African country [32]. The study was from Uganda and included 600 participants. There were no studies from Middle or South American countries or Pacific Island countries and Australia. The studies included in this review focused mostly on high-income countries, thus making the conclusions and generalizations weaker in terms of applying them to Global South nations.

### Conclusions

This review suggests that there should be great concern about the volume of misinformation being spread and the association between COVID-19 vaccine misinformation and vaccine hesitancy. Many studies have shown that there is a link between misinformation on social media and COVID-19 vaccine hesitancy. However, there is a need to examine this effect using a more robust experimental design to assess this effect. It is possible to conduct more experimental studies in an ethical manner in a laboratory setting; for instance, a study to see whether people are able to distinguish between false and true information and how they do so. Such a study would, of course, have to be based on informed consent and be approved by an ethics committee. It is also possible to improve observational studies that extract data from social media by gathering more representative data (eg, including data from several social media platforms, different audiences, several languages, and covering longer periods). There are many types of misinformation that are spread on social media platforms, and to prevent these myths from taking hold, health authorities should openly address and discuss these false claims with both cultural and religious awareness in mind. This review showed that a greater variation in studies is needed when it comes to both social media platforms and geographic location. We only found one study that mentioned misinformation about autism and COVID-19 vaccines, but taking the history of autism and the antivaxx community into account, we believe that this an issue that should be given attention in future research.

Although some major tech companies have taken steps to prevent misinformation, more action is needed to stop this infodemic. One valid approach proposed for infodemic management is first information monitoring (infoveillance); second, to enhance and build eHealth literacy and science literacy capacity; third, to encourage quality improvement processes such as fact-checking and peer review; and finally, to encourage accurate and timely knowledge translation [80].

Misinformation about COVID-19 vaccines is still thriving on social media platforms. However, this undertaking represents a balance between people’s right to speak their minds and strategies to counter the spread of misinformation.

## Appendix

Multimedia Appendix 1 Documentation of systematic literature search.

Multimedia Appendix 2 Thematic analysis.

Multimedia Appendix 3 Assessment of risk of bias.

Multimedia Appendix 4 Grading of Recommendations, Development, and Evaluation (GRADE) scores from the GRADE handbook for quality of evidence.

Multimedia Appendix 5 List of excluded full-text articles.

## Abbreviations

GRADE: Grading of Recommendations Assessment, Development and Evaluation
JBI: Joanna Briggs Institute
MMR: measles, mumps, and rubella
PRISMA: Preferred Reporting Items for Systematic Reviews and Meta-Analyses
WHO: World Health Organization

## Data Availability

All relevant data are available in this paper and its supplementary files. Correspondence and requests for additional material should be addressed to the corresponding author (IS).
