# Supplementary material for: Misinformation About COVID-19 Vaccines on Social Media: Rapid Review
Source: J Med Internet Res. 2022 Aug 4;24(8):e37367. doi: 10.2196/37367 (PMC9359307; doi:10.2196/37367)
Supplement: Multimedia Appendix 1 [file jmir_v24i8e37367_app1.pdf]

# Multimedia Appendix 1. Documentation of Systematic Literature Search

| | |
|---|---|
| **Title:** | Misinformation in social media on covid-19 vaccine |
| **Databases:** | Scopus, PsycInfo (OVID), ERIC (EBSCO), Embase (OVID), PubMed, Cochrane library, Cochrane COVID-19 Study register. |
| **Ordered from:** | Ingjerd Skafle<br>Østfold University College, Faculty of Health, Welfare and Organisation<br>Email: ingjerd.skafle@hiof.no, Phone: +47 696 08 654 |
| **Responsible librarian:** | Kjell Erik Johnsen<br>Østfold University College, The library – Campus Halden<br>Email: kjell.e.johnsen@hiof.no, Phone: +47 696 08 065 |
| **Date of delivery** | The EndNote-library with all references (duplicates included) was made and emailed 2021.09.09.<br>This documentation was made and emailed 2021.10.05. |

| **Database and date** | **Search fields** | **Search string (and limiters or expanders if applied)** | **Numbers of matches** |
|---|---|---|---|
| Scopus, 2021.09.09 | Title, Abstract | (TITLE ((misinformation OR disinformation OR information) AND ("social media" OR facebook OR twitter OR instagram OR whatsapp OR telegram OR tumblr OR pinterest OR youtube OR vkontakte OR snapchat OR tiktok OR weibo OR wechat OR reddit) AND (covid* OR corona* OR "Sars-CoV-2" OR pandemic) AND (vaccine* OR vaccination*)) OR ABS ((misinformation OR disinformation OR information) AND ("social media" OR facebook OR twitter OR instagram OR whatsapp OR telegram OR tumblr OR pinterest OR youtube OR vkontakte OR snapchat OR tiktok OR weibo OR wechat OR reddit) AND (covid* OR corona* OR "Sars-CoV-2" OR pandemic) AND (vaccine* OR vaccination*))) | 201 |
| PsycINFO (OVID), 2021.09.09 | Title, Abstract | ((misinformation OR disinformation OR information) AND ("social media" OR Facebook OR Twitter OR Instagram OR WhatsApp OR Telegram OR Tumblr OR Pinterest OR YouTube OR VKontakte OR Snapchat OR TikTok OR Weibo OR WeChat OR Reddit) AND (covid* OR cORona* OR "Sars-CoV-2" OR pandemic) AND (vaccine* OR vaccination*)).ti. OR ((misinformation OR disinformation OR information) AND ("social media" OR Facebook OR Twitter OR Instagram OR WhatsApp OR Telegram OR Tumblr OR Pinterest OR YouTube OR VKontakte OR Snapchat OR TikTok OR Weibo OR WeChat OR Reddit) AND (covid* OR corona* OR "Sars-CoV-2" OR pandemic) AND (vaccine* OR vaccination*)).ab.<br><br>Limiter: Peer reviewed journals | 12 |
| ERIC (EBSCO), 2021.09.09 | Title, Abstract | TI ( (misinformation OR disinformation OR information) AND ("social media" OR Facebook OR Twitter OR Instagram OR WhatsApp OR Telegram OR Tumblr OR Pinterest OR YouTube OR VKontakte OR Snapchat OR TikTok OR Weibo OR WeChat OR Reddit) AND (covid* OR corona* OR "Sars-CoV-2" OR pandemic) AND (vaccine* OR vaccination*) ) OR AB ( (misinformation OR disinformation OR information) AND ("social media" OR Facebook OR Twitter OR Instagram OR WhatsApp OR Telegram OR Tumblr OR Pinterest OR YouTube OR VKontakte OR Snapchat OR TikTok OR Weibo OR WeChat OR Reddit) AND (covid* OR corona* OR "Sars-CoV-2" OR pandemic) AND (vaccine* OR vaccination*) )<br><br>Limiter: Peer reviewed.<br><br>Expander: Apply equivalent subjects | 0 |
| Embase (OVID), 2021.09.09 | Title, Abstract | ((misinformation OR disinformation OR information) AND ("social media" OR Facebook OR Twitter OR Instagram OR WhatsApp OR Telegram OR Tumblr OR Pinterest OR YouTube OR VKontakte OR Snapchat OR TikTok OR Weibo OR WeChat OR Reddit) AND (covid* OR corona* OR "Sars-CoV-2" OR pandemic) AND (vaccine* OR vaccination*)).ti. OR ((misinformation OR disinformation OR information) AND ("social media" OR Facebook OR Twitter OR Instagram OR WhatsApp OR Telegram OR Tumblr OR Pinterest OR YouTube OR VKontakte OR Snapchat OR TikTok OR Weibo OR WeChat OR Reddit) AND (covid* OR corona* OR "Sars-CoV-2" OR pandemic) AND (vaccine* OR vaccination*)).ab. | 182 |
| PubMed, 2021.09.09 | Title, Abstract | ("misinformation"[Title/Abstract] OR "disinformation"[Title/Abstract] OR "information"[Title/Abstract]) AND ("social media"[Title/Abstract] OR "Facebook"[Title/Abstract] OR "Twitter"[Title/Abstract] OR "Instagram"[Title/Abstract] OR | 196 |

| | | | |
|---|---|---|---|
| | | "WhatsApp"[Title/Abstract] OR "Telegram"[Title/Abstract] OR "Tumblr"[Title/Abstract] OR "Pinterest"[Title/Abstract] OR "YouTube"[Title/Abstract] OR "VKontakte"[Title/Abstract] OR "Snapchat"[Title/Abstract] OR "TikTok"[Title/Abstract] OR "Weibo"[Title/Abstract] OR "WeChat"[Title/Abstract] OR "Reddit"[Title/Abstract]) AND ("covid*"[Title/Abstract] OR "corona*"[Title/Abstract] OR "Sars-CoV-2"[Title/Abstract] OR "pandemic"[Title/Abstract]) AND ("vaccine*"[Title/Abstract] OR "vaccination*"[Title/Abstract]) | |
| Cochrane Library, 2021.09.09 | Title, Abstract | ((misinformation OR disinformation OR information) AND ("social media" OR Facebook OR Twitter OR Instagram OR WhatsApp OR Telegram OR Tumblr OR Pinterest OR YouTube OR VKontakte OR Snapchat OR TikTok OR Weibo OR WeChat OR Reddit) AND (covid* OR corona* OR "Sars-CoV-2" OR pandemic) AND (vaccine* OR vaccination*)):ti OR ((misinformation OR disinformation OR information) AND ("social media" OR Facebook OR Twitter OR Instagram OR WhatsApp OR Telegram OR Tumblr OR Pinterest OR YouTube OR VKontakte OR Snapchat OR TikTok OR Weibo OR WeChat OR Reddit) AND (covid* OR corona* OR "Sars-CoV-2" OR pandemic) AND (vaccine* OR vaccination*)):ab<br><br>Expander: Word variations have been searched | 8 |
| Cochrane COVID-19 Study register, 2021.09.09 | Not applicable | (misinformation OR disinformation OR information) and ("social media" OR Facebook OR Twitter OR Instagram OR WhatsApp OR Telegram OR Tumblr OR Pinterest OR YouTube OR VKontakte OR Snapchat OR TikTok OR Weibo OR WeChat OR Reddit) and (covid* OR corona* OR "Sars-CoV-2" OR pandemic) and (vaccine* OR vaccination*) | 158 |
| | | Total number of references (duplicates included) | 757 |
