# Supplementary material for: Misinformation About COVID-19 Vaccines on Social Media: Rapid Review
Source: J Med Internet Res. 2022 Aug 4;24(8):e37367. doi: 10.2196/37367 (PMC9359307; doi:10.2196/37367)
Supplement: Multimedia Appendix 2 [file jmir_v24i8e37367_app2.pdf]

Multimedia Appendix 2. Thematic Analysis

Theme	Codes	Data Extracts
Conspiracies	• Technology-related<br>• Secret society<br>• Hidden power structures<br>• Secret research<br>• Racism<br>• Corrupt elites<br>• The Deep State	• COVID-19 vaccination is a cover for a plan devised by Bill Gates to implant trackable microchips to control people<br>• COVID-19 vaccines contain a microchip through which biometric data could be collected, and large businesses could send signals to the chips using 5G networks<br>• Part of a new world order to control future populations<br>• The pharmaceutical industries lobbies to kill the elderly and leave the young with Bell's syndrome<br>• Bill Gates and Anthony Fauci had instigated measures (i.e., microchips and enzymes in the vaccine) to control the population through the administration of the COVID-19 vaccine<br>• The vaccines are created for the profit of pharmaceutical companies<br>• COVID-19 is man-made to enforce vaccination<br>• Race extermination conspiracy that claims that the vaccine was created to kill off [people of color] POC.<br>• "Mark of the beast" reference to the New Testament, i.e. those getting the vaccine will be followers of Satan.<br>• Governments and certain powerful individuals "planned" this health crisis to vaccinate children without parental consent as part of the new world order to control future populations<br>• Evangelical hubs posted conspiracy theories about Bill Gates and China
**Theme**	**Codes**	**Data Extracts**
Vaccine Development	• Research/procedural scepticism<br>• Content of vaccines<br>• Vaccine hoax	• Crucial trials in the vaccine development were skipped<br>• The vaccine contains pigs<br>• The vaccine contains aborted children<br>• The vaccine has existed before the COVID-19 epidemic<br>• Contains poison<br>• COVID-19 vaccines are poison and the mRNA technology has not been tested yet and is harmful

Theme	Codes	Data Extracts
Medical Misinformation	• The harms the vaccines can do to your body and mental health.<br><br>• Vaccine side-effects	• The vaccine may lead to infertility<br>• Cause chronic illness<br>• Change DNA<br>• Adverse effects leading to genocide<br>• Can cause physical deformities<br>• Can give mental illness<br>• The immune system is stronger than the vaccines<br>• The vaccines are poisonous<br>• The daughter of the Russian president had died after receiving the second dose of the COVID-19 vaccine<br>• Children and soldiers dying after receiving the vaccine in multiple countries<br>• MRNA can do/mean literally anything from protecting you from COVID to sterilization all the way into making you autistic
