# Supplementary material for: Misinformation About COVID-19 Vaccines on Social Media: Rapid Review
Source: J Med Internet Res. 2022 Aug 4;24(8):e37367. doi: 10.2196/37367 (PMC9359307; doi:10.2196/37367)
Supplement: Multimedia Appendix 3 [file jmir_v24i8e37367_app3.pdf]

Multimedia Appendix 3. Assessment of Risk of Bias. The Joanna Briggs Institute (JBI) Critical Appraisal tool. Checklist for Analytical Cross-Sectional Studies

Studies	Were the criteria for inclusion in the sample clearly defined?	Were the study subjects and the setting described in detail?	Was the exposure measured in a valid and reliable way?	Were objective, standard criteria used for measurement of the condition?	Were confounding factors identified?	Were strategies to deal with confounding factors stated?	Were the outcomes measured in a valid and reliable way?	Was appropriate statistical analysis used?	Total number of yes %*	Risk of Bias**
Alibrahim & Awad[1]	Yes	Yes	Yes	NA	Yes	Yes	Yes	Yes	100 %	Low
Alliheibi[2]	NA	NA	NA	NA	No	No	Yes	NA	33 %	High
Allington et al[3]	Yes	Yes	Yes	NA	Yes	Yes	Yes	Yes	100 %	Low
Aloweidi et al[4]	Yes	Yes	Yes	NA	Yes	Yes	Yes	Yes	100 %	Low
Baines et al[5]	NA	NA	NA	NA	No	No	Yes	NA	33 %	High
Basch et al[6]	NA	NA	NA	NA	No	No	Yes	NA	33 %	High
Berry et al[7]	Yes	No	Yes	NA	No	No	Yes	NA	50 %	Moderate
Bhagianadh & Arora[8]	Yes	Yes	Yes	NA	Yes	Yes	Yes	Yes	100 %	Low
Boucher et al[9]	NA	NA	NA	NA	No	No	Yes	NA	33 %	High
Brodiziak et al[10]	Yes	Yes	Yes	NA	Yes	Yes	Yes	Yes	100 %	Low
Chadwick et al[11]	Yes	Yes	Yes	NA	Yes	Yes	Yes	Yes	100 %	Low
Chan et al[12]	NA	NA	NA	NA	Yes	Yes	Yes	Yes	100 %	Low
Choudhary et al[13]	No	Yes	NA	NA	NA	NA	NA	Yes	67 %	Moderate
Costantino et al[14]	Yes	Yes	Yes	NA	No	No	Yes	Yes	71 %	Low
Chriss et al[15]	NA	NA	NA	NA	No	No	Yes	NA	33 %	High
Doyno et al[16]	NA	NA	NA	NA	NA	NA	NA	NA	0	NA
Ebrahimi et al[17]	Yes	Yes	Yes	NA	Yes	Yes	Yes	Yes	100 %	Low
El-Far Cardo et al[18]	Yes	Yes	Yes	NA	No	No	Yes	No	57 %	Moderate
Guntuku et al[19]	NA	NA	NA	NA	No	No	Yes	Yes	50 %	Moderate
Hernández-García et al[20]	NA	NA	NA	NA	No	No	Yes	Yes	50 %	Moderate
Herrera-Peco et al[21]	NA	NA	NA	NA	No	No	Yes	NA	33 %	High
Herrera-Peco et al[22]	Yes	NA	NA	NA	NA	NA	Yes	Yes	100 %	Low
Hughes et al[23]	Yes	NA	NA	NA	NA	NA	Yes	NA	100 %	Low
Islam et al[24]	NA	NA	NA	NA	No	No	Yes	Yes	50 %	Moderate
Jennings et al[25]	Yes	Yes	Yes	NA	Yes	Yes	Yes	No	86 %	Low
Kanyike et al[26]	Yes	Yes	Yes	NA	Yes	Yes	Yes	Yes	100 %	Low
Karabela et al[27]	Yes	Yes	Yes	NA	Yes	Yes	Yes	Yes	100 %	Low

Knights et al[28]	Yes	Yes	Yes	NA	No	No	Yes	NA	67 %	Moderate
Kwok et al[29]	NA	NA	NA	NA	No	No	Yes	Yes	50 %	Moderate
Larrondo-Ureta[30]	Yes	Yes	NA	NA	NA	NA	NA	Yes	100 %	Low
Liu & Liu[31]	NA	NA	NA	NA	NA	NA	Yes	Yes	100 %	Low
Melton et al[32]	No	No	NA	NA	No	No	Yes	Yes	33 %	High
Park et al[33]	Yes	Yes	Yes	NA	Yes	Yes	Yes	Yes	100 %	Low
Pascual-Ferra[34]	NA	NA	NA	NA	No	No	No	Yes	25 %	High
Petravic et al[35]	Yes	Yes	Yes	NA	Yes	Yes	Yes	Yes	100 %	Low
Rotolo et al[36]	NA	NA	NA	NA	No	No	Yes	NA	33 %	High
Sallam et al[37]	Yes	Yes	Yes	NA	Yes	Yes	Yes	Yes	100 %	Low
Sallam et al[38]	Yes	Yes	Yes	NA	Yes	Yes	Yes	Yes	100 %	Low
Savolainen[39]	NA	NA	NA	NA	No	No	Yes	No	25 %	High
Sharevski & Gover[40]	Yes	Yes	Yes	NA	Yes	Yes	Yes	Yes	100 %	Low
Sobkowicz & Sobkowicz[41]	NA	NA	NA	NA	No	Yes	Yes	Yes	75 %	Low
Thelwall et al[42]	NA	NA	NA	NA	No	No	No	Yes	25 %	High
Wawrzuta et al[43]	NA	NA	NA	NA	No	No	Yes	NA	33 %	High
Zhang et al[44]	Yes	Yes	Yes	NA	Yes	Yes	Yes	Yes	100 %	Low
Zhang et al[45]	Yes	Yes	Yes	NA	Yes	Yes	Yes	Yes	100 %	Low

*Y=Yes; N= No; NA= Not Applicable

** Low risk of bias> 70%; Moderate risk of bias 40-70%; High risk of bias <40%. The percentage was calculated according to have many “yes” each study got relative to the applicable items.

---

1 Alibrahim J, Awad A. COVID-19 vaccine hesitancy among the public in Kuwait: A cross-sectional survey. *Int J Environ* 2021;**18**(16).

2 Alliheibi FM, Omar A, Al-Horais N. Opinion Mining of Saudi Responses to COVID-19 Vaccines on Twitter: A Computational Linguistic Approach. *Int J Adv Comput Sci Appl* 2021;**12**(6):72-8.

3 Allington D, McAndrew S, Moxham-Hall V, Duffy B. Coronavirus conspiracy suspicions, general vaccine attitudes, trust and coronavirus information source as predictors of vaccine hesitancy among UK residents during the COVID-19 pandemic. *Psychol Med*. 2021; 1-12.

4 Aloweidi A, Bsisu I, Suleiman A, Abu-Halaweh S, Almustafa M, Aqel M, et al. Hesitancy towards covid-19 vaccines: An analytical cross–sectional study. *Int J Environ* 2021;**18**(10).

5 Baines A, Ittefaq M, Abwao M. #Scamdemic, #Plandemic, or #Scaredemic: What Parler Social Media Platform Tells Us about COVID-19 Vaccine. *Vaccines* (Basel). 2021; **9**(5): 421. https://doi.org/10.3390/vaccines9050421.

6 Basch CH, Meleo-Erwin Z, Fera J, Jaime C, Basch CE. A global pandemic in the time of viral memes: COVID-19 vaccine misinformation and disinformation on TikTok. *Hum Vaccin Immunother*. 2021; **17**(8):2373-2377. doi: 10.1080/21645515.2021.1894896.

7 Berry SD, Johnson KS, Myles L, Herndon L, Montoya A, Fashaw S, et al. Lessons learned from frontline skilled nursing facility staff regarding COVID-19 vaccine hesitancy. *J Am Geriatr Soc* 2021; **69**(5):1140-6.

[8] Bhagianadh D, Arora K. COVID-19 Vaccine Hesitancy Among Community-Dwelling Older Adults: The Role of Information Sources. *J Appl Gerontol* 2021; **41**(1):4-11. doi: 10.1177/07334648211037507.

[9] Boucher JC, Cornelson K, Benham JL, Fullerton MM, Tang T, Constantinescu C, et al. Analyzing Social Media to Explore the Attitudes and Behaviors Following the Announcement of Successful COVID-19 Vaccine Trials: Infodemiology Study. *JMIR Infodemiology* 2021;**1**(1):e28800.

[10] Brodziak A, Sigorski D, Osmola M, et al. Attitudes of Patients with Cancer towards Vaccinations—Results of Online Survey with Special Focus on the Vaccination against COVID-19. *Vaccines*. 2021; **9**(5):411. https://doi.org/10.3390/vaccines9050411.

[11] Chadwick A, Kaiser J, Vaccari C, Freeman D, Lambe S, Loe BS, et al. Online Social Endorsement and Covid-19 Vaccine Hesitancy in the United Kingdom. *Social Media and Society* 2021;**7**(2).

[12] Chan C, Sounderajah V, Daniels E, Acharya A, Clarke J, Yalamanchili S, et al. The reliability and quality of youtube videos as a source of public health information regarding COVID-19 vaccination: Cross-sectional study. *JMIR Public Health* Surveill 2021;**7**(7).

[13] Choudhary K, Garapati P, Prasad SR, Murti K, Singh S, Kumar N. COVID-19 vaccine hesitancy assessment among the educated Indian population: An online survey. *Int J Pharm Res.* 2021;**13**(3):1358-63.

[14] Costantino C, Gori D, Alba D, Cimino L, Conforto A, Tomasello F, et al. Willingness to receive covid-19 vaccination in costumers accessing community pharmacies in the province of Palermo, Italy. *EuroMediterranean Biomed J* 2021;**16**(19):80-4.

[15] Criss S, Nguyen TT, Norton S, et al. Advocacy, Hesitancy, and Equity: Exploring U.S. Race-Related Discussions of the COVID-19 Vaccine on Twitter. *Int J Environ Res Public Health*. 2021;**18**(11):5693. doi:10.3390/ijerph18115693.

[16] Doyno CR, Fitzgerald JM, White CM, Sobieraj DM, Zacchera M. (in press). Innovative partnership in Connecticut to expand health professional eligibility to administer COVID-19 vaccines. Journal of the American Pharmacists Association. *J Am Pharm Assoc* 2021.

[17] Ebrahimi OV, Johnson MS, Ebling S, Amundsen OM, Halsoy O, Hoffart A, et al. Risk, Trust, and Flawed Assumptions: Vaccine Hesitancy During the COVID-19 Pandemic. *Public Health Front*. 2021;**9**: 700213.

[18] El-Far Cardo A, Kraus T, Kaifie A. Factors That Shape People's Attitudes towards the COVID-19 Pandemic in Germany-The Influence of MEDIA, Politics and Personal Characteristics. *Int J Environ Res Public Health* 2021;**18**(15).

[19] Guntuku SC, Buttenheim AM, Sherman G, Merchant RM. Twitter discourse reveals geographical and temporal variation in concerns about COVID-19 vaccines in the United States. *Vaccine* 2021; **39**(30):4034-8.

[20] Hernández-García I, Gascón-Giménez I, Gascón-Giménez A, Giménez-Júlvez T. Information in Spanish on YouTube about Covid-19 vaccines. *Hum Vaccin Immunother* 2021:1-6.

[21] Herrera-Peco I, Jiménez-Gómez B, Romero Magdalena CS, Deudero JJ, García-Puente M, Benítez De Gracia E, et al. Antivaccine movement and covid-19 negationism: A content analysis of spanish-written messages on twitter. Vaccines. 2021;9(6).

[22] Herrera-Peco I, Ruiz Núñez C, Jiménez-Gómez B, Romero-Magdalena CS, Benítez De Gracia E. COVID-19 and vaccination: analysis of public institution's role in information spread through Twitter. *Revista espanola de salud publica* 2021;95.

[23] Hughes B, Miller-Idriss C, Piltch-Loeb R, Goldberg B, White K, Criezis M, et al. Development of a codebook of online anti-vaccination rhetoric to manage covid-19 vaccine misinformation. *Int J Environ Res* 2021;**18**(14).

[24] Islam MS, Kamal AM, Kabir A, Southern DL, Khan SH, Hasan SMM, et al. COVID-19 vaccine rumors and conspiracy theories: The need for cognitive inoculation against misinformation to improve vaccine adherence. *PLoS One* 2021;**16**(5):e0251605.

[25] Jennings W, Stoker G, Bunting H, Valgarðsson VO, Gaskell J, Devine D, et al. Lack of Trust, Conspiracy Beliefs, and Social Media Use Predict COVID-19 Vaccine Hesitancy. *Vaccines* (Basel). 2021;**9**(6).

[26] Kanyike AM, Olum R, Kajjimu J, Ojilong D, Akech GM, Nassozi DR, et al. Acceptance of the coronavirus disease-2019 vaccine among medical students in Uganda. *Trop Med Health*. 2021;**49**(1).

[27] Karabela ŞN, Coşkun F, Hoşgör H. Investigation of the relationships between perceived causes of COVID-19, attitudes towards vaccine and level of trust in information sources from the perspective of Infodemic: the case of Turkey. *BMC Public Health* 2021; **21**(1):1195. doi: 10.1186/s12889-021-11262-1.
[28] Knights F, Carter J, Deal A, Crawshaw AF, Hayward SE, Jones L, et al. Impact of COVID-19 on migrants' access to primary care and implications for vaccine roll-out: A national qualitative study. British Journal of General Practice. 2021;**71**(709):E583-E95.
[29] Kwok SWH, Vadde SK, Wang G. Tweet Topics and Sentiments Relating to COVID-19 Vaccination Among Australian Twitter Users: Machine Learning Analysis. *J Med Internet Res* 2021;**23**(5):e26953.
[30] Larrondo-Ureta A, Fernández SP, Morales-I-gras J. Disinformation, vaccines, and covid-19. Analysis of the infodemic and the digital conversation on twitter. *Revista Latina de Comunicacion Social* 2021; **79**:1-18.
[31] Liu S, Liu J. Understanding Behavioral Intentions Toward COVID-19 Vaccines: Theory-Based Content Analysis of Tweets. *J Med Internet Res* 2021;**23**(5):e28118.
[32] Melton CA, Olusanya OA, Ammar N, Shaban-Nejad A. Public sentiment analysis and topic modeling regarding COVID-19 vaccines on the Reddit social media platform: A call to action for strengthening vaccine confidence. *J Infect Public Health* 2021;**14**(10):1505-1512. doi:10.1016/j.jiph.2021.08.010.
[33] Park S, Massey PM, Stimpson JP. Primary Source of Information About COVID-19 as a Determinant of Perception of COVID-19 Severity and Vaccine Uptake: Source of Information and COVID-19. *J Gen Intern Med* 2021; 1-8.
[34] Pascual-Ferrá P, Alperstein N, Barnett DJ. A Multi-platform Approach to Monitoring Negative Dominance for COVID-19 Vaccine-Related Information Online [published online ahead of print, 2021 May 3]. *Disaster Med Public Health Prep*. 2021;1-24. doi:10.1017/dmp.2021.136.
[35] Petravić L, Arh R, Gabrovec T, et al. Factors Affecting Attitudes towards COVID-19 Vaccination: An Online Survey in Slovenia. Vaccines 2021; **9**(3):247. doi: 10.3390/vaccines9030247.
[36] Rotolo SM, Jain S, Dhaon S, Dokhanchi JK, Kalata E, Shah T, et al. (in press). A coordinated strategy to develop and distribute infographics addressing COVID-19 vaccine hesitancy and misinformation. *J Am Pharm Assoc (2003)* 2021.
[37] Sallam M, Dababseh D, Eid H, Hasan H, Taim D, Al-Mahzoum K, et al. Low COVID-19 Vaccine Acceptance Is Correlated with Conspiracy Beliefs among University Students in Jordan. *Int J Environ Res Public Health* 2021;**18**(5).
[38] Sallam M, Dababseh D, Eid H, et al. High Rates of COVID-19 Vaccine Hesitancy and Its Association with Conspiracy Beliefs: A Study in Jordan and Kuwait among Other Arab Countries. *Vaccines (Basel)*. 2021;**9**(1):42. Published 2021 Jan 12. doi:10.3390/vaccines9010042.
[39] Savolainen R. Assessing the credibility of COVID-19 vaccine mis/disinformation in online discussion. *Journal of Information Science* 2021. doi:10.1177/01655515211040653.
[40] Sharevski F, Gover D. Two Truths and a Lie: Exploring Soft Moderation of COVID-19 Misinformation with Amazon Alexa. In The 16th International Conference on Availability, Reliability and Security (ARES 2021). Association for Computing Machinery, New York, NY, USA, Article 79, 1–9. DOI:https://doi.org/10.1145/3465481.3470017.
[41] Sobkowicz P, Sobkowicz A. Agent Based Model of Anti-Vaccination Movements: Simulations and Comparison with Empirical Data. *Vaccines (Basel)* 2021;**9**(8):809. Published 2021 Jul 21. doi:10.3390/vaccines9080809.
[42] Thelwall M, Kousha K, Thelwall S. Covid-19 vaccine hesitancy on English-language Twitter. *Profesional de la Informacion* 2021;**30**(2). e300212. https://doi.org/10.3145/epi.2021.mar.12.
[43] Wawrzuta D, Jaworski M, Gotlib J, Panczyk M. What arguments against COVID-19 vaccines run on facebook in poland: Content analysis of comments. *Vaccines* 2021;9(5).
[44] Zhang KC, Fang Y, Cao H, Chen H, Hu T, Chen Y, et al. Behavioral intention to receive a COVID-19 vaccination among Chinese factory workers: Cross-sectional online survey. *JMIR* 2021;**23**(3).
[45] Zhang KC, Fang Y, Cao H, Chen H, Hu T, Chen YQ, et al. Parental acceptability of COVID-19 vaccination for children under the age of 18 years: Cross-sectional online survey. JMIR Pediatr Parent 2020;**3**(2).
