# Supplementary material for: Misinformation About COVID-19 Vaccines on Social Media: Rapid Review
Source: J Med Internet Res. 2022 Aug 4;24(8):e37367. doi: 10.2196/37367 (PMC9359307; doi:10.2196/37367)
Supplement: Multimedia Appendix 4 [file jmir_v24i8e37367_app4.pdf]

Multimedia Appendix 4. GRADE Scores from the GRADE handbook for grading quality of evidence and strength of recommendations.

Studies	GRADE Score*	Sampling	Bias/limitations discussed	Study design/method
Alibrahim & Awad[1]	1	Non-random sampling	Yes	Observational/survey on vaccine hesitancy
Alliheibi[2]	1	Sample described	No	Observational/content analysis of tweets regarding attitudes to vaccination
Allington et al[3]	2	Sample representative	Yes	Observational/survey on predictors of vaccine hesitancy
Aloweidi et al[4]	1-2	Sampling online/self-selected	Yes	Observational/survey on vaccine hesitancy
Baines et al[5]	2	Random sample	Yes	Observational/text analysis of Parler content
Basch et al[6]	2	Sampling based on numbers	Yes	Observational/analysis of popular YouTube videos on vaccines
Berry et al[7]	1	Non-random sample	No	Observational/group interviews on vaccine concerns
Bhagianadh & Arora[8]	2	Sample well described	Yes	Observational/survey on information sources
Boucher et al[9]	2	Sample described	Yes	Observational/content analysis of tweets by machine learning
Brodiziak et al[10]	1-2	Sample non-random (?)	Briefly discussed	Observational/survey on opinions on vaccination
Chadwick et al[11]	2	Representative sample	Yes	Observational/survey 'media diets'
Chan et al[12]	2	Sampling acceptably described	Yes	Observational, content analysis of YouTube videos
Choudhary et al[13]	1	Non-random/online sampling	No	Observational/survey
Costantino et al[14]	1	Non-random (self-selected)	Briefly discussed	Observational/survey
Chriss et al[15]	1	Sample described	Yes	Observational/content analysis of tweets
Doyno et al[16]	1	Sample described<br><br>No control group	No	Interventional study/description of a training program and videos used to train vaccinators
Ebrahimi et al[17]	1-2	Sample non-random/self-selected	Yes	Observational/survey on vaccine hesitancy
El-Far Cardo et al[18]	1-2	Sample non-random (online/self-selected)	Yes	Observational/survey
Guntuku et al[19]	1-2	Sample described	Yes, briefly	Observational/content analysis of tweets on vaccines
Hernández-García et al[20]	2	Sample described	Yes	Observational/content analysis of YouTube videos on vaccines
Herrera-Peco et al[21]	1-2	Sample described	Yes	Observational/content analysis of antivax tweets
Herrera-Peco et al[22]	1-2	Sample described	Yes	Observational/content analysis of tweets from institutions
Hughes et al[23]	1-2	Non-random/purposive sampling	Bias/limitations discussed	Observational/qualitative content analysis of antivax rhetoric on social media/film Sentiment/'code book' of rhetorics
Islam et al[24]	1	Unclear sampling	Bias poorly discussed. Poorly conducted	Google, social media/observational
Jennings et al[25]	2	Sample well defined	Yes	Survey/observational
Kanyike et al[26]	1	Non-random sample (self-selected)	Yes, briefly	Observational/survey
Karabela et al[27]	1	Sample non-random (self-recruited)	No	Observational/survey
Knights et al[28]	1-2	Non-random sampling	Yes	Observational study
Kwok et al[29]	2	Sample described	Yes	Observational study of tweets
Larrondo-Ureta[30]	1	Sample described	Bias/limitations poorly discussed	Observational Qualitative content analysis of tweets
Liu & Liu[31]	2	Sampling described well	Yes	Observational Qualitative content analysis of Twitter posts

Melton et al[32]	1-2	Unclear sampling	Yes	Observational/sentiment analysis of Reddit posts
Park et al[33]	2	Sampling clear	Yes	Observational/survey
Pascual-Ferra[34]	1	Unclear design	Yes	Observational/social media trends vs. 'adverse events'
Petravic et al[35]	1-2	Unclear sampling/online	Yes	Observational/survey
Rotolo et al[36]	2-3	Infografics created and spread on social media, reach evaluated	Yes	Intervention study, no randomization
Sallam et al[37]	1-2	Online/unclear sampling	Yes	Observational/survey
Sallam et al[38]	1-2	Online/unclear sampling	Yes	Observational/survey
Savolainen[39]	1	Unclear sampling	No	Observational study of Reddit posts
Sharevski & Gover[40]	3	Clear sampling but self selected (online)	Yes	Intervention study with randomization
Sobkowicz & Sobkowicz[41]	2	Clear sampling	Yes	Social media/observational
Thelwall et al[42]	1	Clear sampling	Bias poorly discussed	Twitter/observational
Wawrzuta et al[43]	1	Sampling unclear	No	Observational/content analysis of Facebook posts/comments
Zhang et al[44]	2	Sampling described	Yes	Observational/online survey of attitudes to vaccination
Zhang et al[45]	2	Sampling described	Yes	Observational/online survey of attitudes to vaccination

***GRADE Scores: 1=very low, 2=low, 3=moderate, 4=high**

References

1 Alibrahim J, Awad A. COVID-19 vaccine hesitancy among the public in Kuwait: A cross-sectional survey. *Int J Environ* 2021;**18**(16).

2 Alliheibi FM, Omar A, Al-Horais N. Opinion Mining of Saudi Responses to COVID-19 Vaccines on Twitter: A Computational Linguistic Approach. *Int J Adv Comput Sci Appl* 2021;**12**(6):72-8.

3 Allington D, McAndrew S, Moxham-Hall V, Duffy B. Coronavirus conspiracy suspicions, general vaccine attitudes, trust and coronavirus information source as predictors of vaccine hesitancy among UK residents during the COVID-19 pandemic. *Psychol Med*. 2021; 1-12.

4 Aloweidi A, Bsisu I, Suleiman A, Abu-Halaweh S, Almustafa M, Aqel M, et al. Hesitancy towards covid-19 vaccines: An analytical cross–sectional study. *Int J Environ* 2021;**18**(10).

5 Baines A, Ittefaq M, Abwao M. #Scamdemic, #Plandemic, or #Scaredemic: What Parler Social Media Platform Tells Us about COVID-19 Vaccine. *Vaccines* (Basel). 2021; **9**(5): 421. https://doi.org/10.3390/vaccines9050421.

6 Basch CH, Meleo-Erwin Z, Fera J, Jaime C, Basch CE. A global pandemic in the time of viral memes: COVID-19 vaccine misinformation and disinformation on TikTok. *Hum Vaccin Immunother*. 2021; **17**(8):2373-2377. doi: 10.1080/21645515.2021.1894896.

7 Berry SD, Johnson KS, Myles L, Herndon L, Montoya A, Fashaw S, et al. Lessons learned from frontline skilled nursing facility staff regarding COVID-19 vaccine hesitancy. *J Am Geriatr Soc* 2021; **69**(5):1140-6.

8 Bhagianadh D, Arora K. COVID-19 Vaccine Hesitancy Among Community-Dwelling Older Adults: The Role of Information Sources. *J Appl Gerontol* 2021; **41**(1):4-11. doi: 10.1177/07334648211037507.

9 Boucher JC, Cornelson K, Benham JL, Fullerton MM, Tang T, Constantinescu C, et al. Analyzing Social Media to Explore the Attitudes and Behaviors Following the Announcement of Successful COVID-19 Vaccine Trials: Infodemiology Study. *JMIR Infodemiology* 2021;**1**(1):e28800.

10 Brodziak A, Sigorski D, Osmola M, et al. Attitudes of Patients with Cancer towards Vaccinations—Results of Online Survey with Special Focus on the Vaccination against COVID-19. *Vaccines*. 2021; **9**(5):411. https://doi.org/10.3390/vaccines9050411.

[11] Chadwick A, Kaiser J, Vaccari C, Freeman D, Lambe S, Loe BS, et al. Online Social Endorsement and Covid-19 Vaccine Hesitancy in the United Kingdom. *Social Media and Society* 2021;**7**(2).

[12] Chan C, Sounderajah V, Daniels E, Acharya A, Clarke J, Yalamanchili S, et al. The reliability and quality of youtube videos as a source of public health information regarding COVID-19 vaccination: Cross-sectional study. *JMIR Public Health* Surveill 2021;**7**(7).

[13] Choudhary K, Garapati P, Prasad SR, Murti K, Singh S, Kumar N. COVID-19 vaccine hesitancy assessment among the educated Indian population: An online survey. *Int J Pharm Res.* 2021;**13**(3):1358-63.

[14] Costantino C, Gori D, Alba D, Cimino L, Conforto A, Tomasello F, et al. Willingness to receive covid-19 vaccination in costumers accessing community pharmacies in the province of Palermo, Italy. *EuroMediterranean Biomed J* 2021;**16**(19):80-4.

[15] Criss S, Nguyen TT, Norton S, et al. Advocacy, Hesitancy, and Equity: Exploring U.S. Race-Related Discussions of the COVID-19 Vaccine on Twitter. *Int J Environ Res Public Health*. 2021;**18**(11):5693. doi:10.3390/ijerph18115693.

[16] Doyno CR, Fitzgerald JM, White CM, Sobieraj DM, Zacchera M. (in press). Innovative partnership in Connecticut to expand health professional eligibility to administer COVID-19 vaccines. Journal of the American Pharmacists Association. *J Am Pharm Assoc* 2021.

[17] Ebrahimi OV, Johnson MS, Ebling S, Amundsen OM, Halsoy O, Hoffart A, et al. Risk, Trust, and Flawed Assumptions: Vaccine Hesitancy During the COVID-19 Pandemic. *Public Health Front*. 2021;**9**: 700213.

[18] El-Far Cardo A, Kraus T, Kaifie A. Factors That Shape People's Attitudes towards the COVID-19 Pandemic in Germany-The Influence of MEDIA, Politics and Personal Characteristics. *Int J Environ Res Public Health* 2021;**18**(15).

[19] Guntuku SC, Buttenheim AM, Sherman G, Merchant RM. Twitter discourse reveals geographical and temporal variation in concerns about COVID-19 vaccines in the United States. *Vaccine* 2021; **39**(30):4034-8.

[20] Hernández-García I, Gascón-Giménez I, Gascón-Giménez A, Giménez-Júlvez T. Information in Spanish on YouTube about Covid-19 vaccines. *Hum Vaccin Immunother* 2021:1-6.

[21] Herrera-Peco I, Jiménez-Gómez B, Romero Magdalena CS, Deudero JJ, García-Puente M, Benítez De Gracia E, et al. Antivaccine movement and covid-19 negationism: A content analysis of spanish-written messages on twitter. Vaccines. 2021;9(6).

[22] Herrera-Peco I, Ruiz Núñez C, Jiménez-Gómez B, Romero-Magdalena CS, Benítez De Gracia E. COVID-19 and vaccination: analysis of public institution's role in information spread through Twitter. *Revista espanola de salud publica* 2021;95.

[23] Hughes B, Miller-Idriss C, Piltch-Loeb R, Goldberg B, White K, Criezis M, et al. Development of a codebook of online anti-vaccination rhetoric to manage covid-19 vaccine misinformation. *Int J Environ Res* 2021;**18**(14).

[24] Islam MS, Kamal AM, Kabir A, Southern DL, Khan SH, Hasan SMM, et al. COVID-19 vaccine rumors and conspiracy theories: The need for cognitive inoculation against misinformation to improve vaccine adherence. *PLoS One* 2021;**16**(5):e0251605.

[25] Jennings W, Stoker G, Bunting H, Valgarðsson VO, Gaskell J, Devine D, et al. Lack of Trust, Conspiracy Beliefs, and Social Media Use Predict COVID-19 Vaccine Hesitancy. *Vaccines* (Basel). 2021;**9**(6).

[26] Kanyike AM, Olum R, Kajjimu J, Ojilong D, Akech GM, Nassozi DR, et al. Acceptance of the coronavirus disease-2019 vaccine among medical students in Uganda. *Trop Med Health*. 2021;**49**(1).

[27] Karabela ŞN, Coşkun F, Hoşgör H. Investigation of the relationships between perceived causes of COVID-19, attitudes towards vaccine and level of trust in information sources from the perspective of Infodemic: the case of Turkey. *BMC Public Health* 2021; **21**(1):1195. doi: 10.1186/s12889-021-11262-1.

[28] Knights F, Carter J, Deal A, Crawshaw AF, Hayward SE, Jones L, et al. Impact of COVID-19 on migrants' access to primary care and implications for vaccine roll-out: A national qualitative study. British Journal of General Practice. 2021;**71**(709):E583-E95.

[29] Kwok SWH, Vadde SK, Wang G. Tweet Topics and Sentiments Relating to COVID-19 Vaccination Among Australian Twitter Users: Machine Learning Analysis. *J Med Internet Res* 2021;**23**(5):e26953.

[30] Larrondo-Ureta A, Fernández SP, Morales-I-gras J. Disinformation, vaccines, and covid-19. Analysis of the infodemic and the digital conversation on twitter. *Revista Latina de Comunicacion Social* 2021; **79**:1-18.

[31] Liu S, Liu J. Understanding Behavioral Intentions Toward COVID-19 Vaccines: Theory-Based Content Analysis of Tweets. *J Med Internet Res* 2021;**23**(5):e28118.

[32] Melton CA, Olusanya OA, Ammar N, Shaban-Nejad A. Public sentiment analysis and topic modeling regarding COVID-19 vaccines on the Reddit social media platform: A call to action for strengthening vaccine confidence. *J Infect Public Health* 2021;**14**(10):1505-1512. doi:10.1016/j.jiph.2021.08.010.

[33] Park S, Massey PM, Stimpson JP. Primary Source of Information About COVID-19 as a Determinant of Perception of COVID-19 Severity and Vaccine Uptake: Source of Information and COVID-19. *J Gen Intern Med* 2021; 1-8.

[34] Pascual-Ferrá P, Alperstein N, Barnett DJ. A Multi-platform Approach to Monitoring Negative Dominance for COVID-19 Vaccine-Related Information Online [published online ahead of print, 2021 May 3]. *Disaster Med Public Health Prep*. 2021;1-24. doi:10.1017/dmp.2021.136.

[35] Petravić L, Arh R, Gabrovec T, et al. Factors Affecting Attitudes towards COVID-19 Vaccination: An Online Survey in Slovenia. Vaccines 2021; **9**(3):247. doi: 10.3390/vaccines9030247.

[36] Rotolo SM, Jain S, Dhaon S, Dokhanchi JK, Kalata E, Shah T, et al. (in press). A coordinated strategy to develop and distribute infographics addressing COVID-19 vaccine hesitancy and misinformation. *J Am Pharm Assoc (2003)* 2021.

[37] Sallam M, Dababseh D, Eid H, Hasan H, Taim D, Al-Mahzoum K, et al. Low COVID-19 Vaccine Acceptance Is Correlated with Conspiracy Beliefs among University Students in Jordan. *Int J Environ Res Public Health* 2021;**18**(5).

[38] Sallam M, Dababseh D, Eid H, et al. High Rates of COVID-19 Vaccine Hesitancy and Its Association with Conspiracy Beliefs: A Study in Jordan and Kuwait among Other Arab Countries. *Vaccines (Basel)*. 2021;**9**(1):42. Published 2021 Jan 12. doi:10.3390/vaccines9010042.

[39] Savolainen R. Assessing the credibility of COVID-19 vaccine mis/disinformation in online discussion. *Journal of Information Science* 2021. doi:10.1177/01655515211040653.

[40] Sharevski F, Gover D. Two Truths and a Lie: Exploring Soft Moderation of COVID-19 Misinformation with Amazon Alexa. In The 16th International Conference on Availability, Reliability and Security (ARES 2021). Association for Computing Machinery, New York, NY, USA, Article 79, 1–9. DOI:https://doi.org/10.1145/3465481.3470017.

[41] Sobkowicz P, Sobkowicz A. Agent Based Model of Anti-Vaccination Movements: Simulations and Comparison with Empirical Data. *Vaccines (Basel)* 2021;**9**(8):809. Published 2021 Jul 21. doi:10.3390/vaccines9080809.

[42] Thelwall M, Kousha K, Thelwall S. Covid-19 vaccine hesitancy on English-language Twitter. *Profesional de la Informacion* 2021;**30**(2). e300212. https://doi.org/10.3145/epi.2021.mar.12.

[43] Wawrzuta D, Jaworski M, Gotlib J, Panczyk M. What arguments against COVID-19 vaccines run on facebook in poland: Content analysis of comments. *Vaccines* 2021;9(5).

[44] Zhang KC, Fang Y, Cao H, Chen H, Hu T, Chen Y, et al. Behavioral intention to receive a COVID-19 vaccination among Chinese factory workers: Cross-sectional online survey. *JMIR* 2021;**23**(3).

[45] Zhang KC, Fang Y, Cao H, Chen H, Hu T, Chen YQ, et al. Parental acceptability of COVID-19 vaccination for children under the age of 18 years: Cross-sectional online survey. JMIR Pediatr Parent 2020;**3**(2).
