# Supplementary material for: Misinformation About COVID-19 Vaccines on Social Media: Rapid Review
Source: J Med Internet Res. 2022 Aug 4;24(8):e37367. doi: 10.2196/37367 (PMC9359307; doi:10.2196/37367)
Supplement: Multimedia Appendix 5 [file jmir_v24i8e37367_app5.pdf]

Multimedia Appendix 5. List of full-text articles excluded, with reasons (n=75)

Not about misinformation: 31

Not about COVID-19 vaccines: 17

Not about social media: 13

Too early in the COVID-19 vaccine development, i.e. not from phase III or IV: 13

Not an empirical study: 1

Study	Reason for exclusion
1. Abu-Farha R, Mukattash T, Itani R, et al. Willingness of Middle Eastern public to receive COVID-19 vaccines. *Saudi Pharm J*. 2021;**29**(7):734-739. doi:10.1016/j.jsps.2021.05.005	Not about misinformation
2. Aci OS, Kackin O, Karaaslan S, Ciydem E. Qualitative examination of the attitudes of healthcare workers in Turkey regarding COVID-19 vaccines. *Int J Nurs Knowl* 2021.	Not about misinformation
3. Al-Marshoudi S, Al-Balushi H, Al-Wahaibi A, et al. Knowledge, Attitudes, and Practices (KAP) toward the COVID-19 Vaccine in Oman: A Pre-Campaign Cross-Sectional Study. *Vaccines (Basel)*. 2021;9(6):602. Published 2021 Jun 4. doi:10.3390/vaccines9060602.	Not about misinformation
4. Aldossari KK, Alharbi MB, Alkahtani SM, Alrowaily TZ, Alshaikhi AM, Twair AA. COVID-19 vaccine hesitancy among patients with diabetes in Saudi Arabia. *Diabetes Metab Syndr*. 2021;**15**(5):102271. doi:10.1016/j.dsx.2021.102271	Not about misinformation<br>.
5. Ali KF, Whitebridge S, Jamal MH, Alsafy M, Atkin SL. Perceptions, Knowledge, and Behaviors Related to COVID-19 Among Social Media Users: Cross-Sectional Study. *J Med Internet Res*. 2020;22(9):e19913. Published 2020 Sep 8. doi:10.2196/19913	Not about the COVID-10 vaccine<br><br>Not about misinformation
6. Basch CE, Basch CH, Hillyer GC, Meleo-Erwin ZC, Zagnit EA YouTube Videos and Informed Decision-Making About COVID-19 Vaccination: Successive Sampling Study. *JMIR Public Health Surveill* 2021;7(5):e28352	Not about misinformation
7. Basch CH, Hillyer GC, Zagnit EA, Basch CE. YouTube coverage of COVID-19 vaccine development: implications for awareness and uptake. *Hum Vaccin Immunother*. 2020;16(11):2582-2585. doi:10.1080/21645515.2020.1790280	Too early in the COVID-19 vaccine development.<br><br>From April 6, 2020<br>Too early in the Covid-19 vaccine development, i.e. not from phase III or IV
8. Bendau A, Plag J, Petzold MB, Ströhle A. COVID-19 vaccine hesitancy and related fears and anxiety. *Int Immunopharmacol*. 2021;97:107724. doi:10.1016/j.intimp.2021.107724	Not about social media
9. Lang R, Benham JL, Atabati O, et al. Attitudes, behaviours and barriers to public health measures for COVID-19: a survey to inform public health messaging. *BMC Public Health*. 2021; (1):765. DOI: 10.1186/s12889-021-10790-0. PMID: 33882896; PMCID: PMC8058588.	Not about COVID-19 vaccine or misinformation
10. Benis A, Seidmann A, Ashkenazi S. Reasons for Taking the COVID-19 Vaccine by US Social Media Users. *Vaccines (Basel)*. 2021;**9**(4):315. Published 2021 Mar 29. doi:10.3390/vaccines9040315	Not about misinformation
11. Berenson AB, Chang M, Hirth JM, Kanukurthy M. Intent to get vaccinated against COVID-19 among reproductive-aged women in Texas. *Hum Vaccin Immunother*. 2021;**17**(9):2914-2918. doi:10.1080/21645515.2021.1918994	Not about social media or misinformation
12. Chaudhary FA, Ahmad B, Khalid MD, Fazal A, Javaid MM, Butt DQ. Factors influencing COVID-19 vaccine hesitancy and acceptance among the Pakistani population. *Hum Vaccin Immunother* 2021;**17**(10):3365-3370. doi:10.1080/21645515.2021.1944743	Not about misinformation
13. Chen T, Rong J, Yang J, Cong G, Li G. Combining Public Opinion Dissemination with Polarization Process Considering Individual Heterogeneity. *Healthcare (Basel)*. 2021;**9**(2):176. Published 2021 Feb 7. doi:10.3390/healthcare9020176	Not about COVID-19 vaccines.<br>Removed during data extraction. Testing out a theory.

14. Costantini H. COVID-19 Vaccine Literacy of Family Carers for Their Older Parents in Japan. *Healthcare (Basel)*. 2021;**9**(8):1038. Published 2021 Aug 12. doi:10.3390/healthcare9081038	Not about misinformation
15. Cotfas LA, Delcea C, Roxin I, Ioanăș C, Gherai DS, Tajariol F. The Longest Month: Analyzing COVID-19 Vaccination Opinions Dynamics from Tweets in the Month following the First Vaccine Announcement. *IEEE Access* 2021;**9**:33203-23. doi: 10.1109/ACCESS.2021.3059821.	Not about misinformation
16. Di Gennaro F, Murri R, Segala FV, et al. Attitudes towards Anti-SARS-CoV2 Vaccination among Healthcare Workers: Results from a National Survey in Italy. *Viruses*. 2021;**13**(3):371. Published 2021 Feb 26. doi:10.3390/v13030371	Not about social media or misinformation
17. Gbashi S, Adebo OA, Doorsamy W, Njobeh PB Systematic Delineation of Media Polarity on COVID-19 Vaccines in Africa: Computational Linguistic Modeling Study. *JMIR Med Inform* 2021;**9**(3):e22916 doi: 10.2196/22916	Not about COVID-19 vaccines or misinformation
18. Germani F, Biller-Andorno N. The anti-vaccination infodemic on social media: A behavioral analysis. *PLoS One*. 2021;16(3):e0247642. Published 2021 Mar 3. doi:10.1371/journal.pone.0247642	Not about COVID-19 vaccines.<br><br>Removed during data extraction. About ani-vaxx in general.
19. Gerts D, Shelley CD, Parikh N, Pitts T, Watson Ross C, Fairchild G, Vaquera Chavez NY, Daughton AR "Thought I'd Share First" and Other Conspiracy Theory Tweets from the COVID-19 Infodemic: Exploratory Study. *JMIR Public Health Surveill* 2021;**7**(4):e26527 doi: 10.2196/26527	Too early in the COVID-19 vaccine development.<br><br>Removed during data extraction due to the dates they collected data (January to early May 2020)
20. Gokhale S. "Monitoring the Perception of Covid-19 Vaccine using Topic Models," *2020 IEEE Intl Conf on Parallel & Distributed Processing with Applications, Big Data & Cloud Computing, Sustainable Computing & Communications, Social Computing & Networking (ISPA/BDCloud/SocialCom/SustainCom)*, 2020, pp. 867-874, doi: 10.1109/ISPA-BDCloud-SocialCom-SustainCom51426.2020.00134.	Too early in the COVID-19 vaccine development.<br><br>Removed during data extraction. Tweets harvested on May 20, 2020
21. Greene C. M.& Murphy G. Quantifying the effects of fake news on behavior: Evidence from a study of COVID-19 misinformation. *J Exp Psychol Appl 2021*. Advance online publication. https://doi.org/10.1037/xap0000371	Not about social media<br><br>Removed during data extraction.
22. Grimes DR. Medical disinformation and the unviable nature of COVID-19 conspiracy theories. *PLoS ONE* 2021; **16**(3): e0245900. https://doi.org/10.1371/journal.pone.0245900	Not about COVID-19 vaccines
23. Harhay MN, Klassen AC, Zaidi H, et al. Living Organ Donor Perspectives and Sources of Hesitancy about COVID-19 Vaccines. *Kidney360*. 2021;**2**(7):1132-1140. doi:10.34067/kid.0002112021	Not about misinformation
24. Hashemi M. Discovering social media topics and patterns in the coronavirus and election era. *J Inf Commun Ethics Soc* 2021. ID: covidwho-1360402	Not about the COVID-19 vaccines
25. Herrera-Peco I, Jiménez-Gómez B, Peña Deudero JJ, Benitez, De Gracia E, Ruiz-Núñez C. Healthcare Professionals' Role in Social Media Public Health Campaigns: Analysis of Spanish Pro Vaccination Campaign on Twitter. *Healthcare (Basel)*. 2021;**9**(6):662. Published 2021 Jun 2. doi:10.3390/healthcare9060662	Not about misinformation
26. Hou Z, Tong Y, Du F, et al. Assessing COVID-19 Vaccine Hesitancy, Confidence, and Public Engagement: A Global Social Listening Study. *J Med Internet Res*. 2021;**23**(6):e27632. Published 2021 Jun 11. doi:10.2196/27632	Too early in the COVID-19 vaccine development.<br><br>Removed during data extraction. The data covered the period from June 13 to July 31, 2020
27. Huynh G, Nguyen TV, Nguyen DD, Lam QM, Pham TN, Nguyen HTN. Knowledge About COVID-19, Beliefs and Vaccination Acceptance Against COVID-19 Among High-Risk People in Ho Chi Minh City, Vietnam. *Infect Drug Resist*. 2021;**14**:1773-1780. Published 2021 May 13. doi:10.2147/IDR.S308446	Not about social media or misinformation

28. Jain J, Saurabh S, Kumar P, et al. COVID-19 vaccine hesitancy among medical students in India. *Epidemiol Infect*. 2021;**149**:e132. Published 2021 May 20. doi:10.1017/S0950268821001205	Not about misinformation
29. Jamison AM, Broniatowski DA, Dredze M, Sangraula A, Smith MC, Quinn SC. Not just conspiracy theories: Vaccine opponents and proponents add to the COVID-19 'infodemic' on Twitter. *Harv Kennedy Sch Misinformation Rev*. 2020;**1**:10.37016/mr-2020-38. doi:10.37016/mr-2020-38	Too early in the COVID-19 vaccine development.<br><br>Examined Tweets from February 2020.
30. Jang H, Rempel E, Roth D, Carenini G, Janjua NZ. Tracking COVID-19 Discourse on Twitter in North America: Infodemiology Study Using Topic Modeling and Aspect-Based Sentiment Analysis. *J Med Internet Res*. 2021;**23**(2):e25431. Published 2021 Feb 10. doi:10.2196/25431	Not about COVID-19 vaccines
31. Janssens U, Kluge S, Marx G, Hermes C, Salzberger B, Karagiannidis C. Einstellung zur Impfung gegen SARS-CoV-2 : Umfrage unter Mitarbeitenden in Krankenhäusern vor und nach Beginn der Impfungen in den deutschen Krankenhäusern [Attitude towards vaccination against SARS-CoV-2 : Survey among employees in hospitals before and after the start of vaccinations in German hospitals]. *Med Klin Intensivmed Notfmed*. 2021;**116**(5):421-430. doi:10.1007/s00063-021-00821-4	Not about social media
32. Kalichman SC, Eaton LA, Earnshaw VA, Brousseau N. Faster than warp speed: early attention to COVD-19 by anti-vaccine groups on Facebook [published online ahead of print, 2021 Apr 9]. *J Public Health (Oxf)*. 2021;fdab093. doi:10.1093/pubmed/fdab093	Too early in the COVID-19 vaccine development.<br><br>Removed during data extraction. Data from March 2020
33. Kolapo, A., Ilesanmi, O., Omoju, T., Odukanmi, O., Akanbi, D., Okediran, J., Balogun, M. Knowledge, Perception, and Sources of Information on COVID-19 among Nigerian Youths in the First Month of the Pandemic. *Journal of Health Sciences & Surveillance System*, 2021; **9**(2): 89-96. doi: 10.30476/jhsss.2020.88288.1140	Not about COVID-19 vaccines
34 Laurencin CT, Valantine H, Yancy C, Jones CP, Bright C. The COVID-19 Vaccine and the Black Community: Addressing the Justified Questions. *J Racial Ethn Health Disparities*. 2021;**8**(4):809-820. doi:10.1007/s40615-021-01082-9	Not about social media.<br><br>Removed during data extraction.
35. Lee H, Noh EB, Park SJ, Nam HK, Lee TH, Lee GR, Nam EW. COVID-19 Vaccine Perception in South Korea: Web Crawling Approach. *JMIR Public Health Surveill* 2021;7(9):e31409. doi: 10.2196/31409	Not about misinformation
36. Li T, Hsu W, Lee ML, Chieu HL, editors. Probabilistic Decision Modeling in Social Networks. Proceedings - International Conference on Tools with Artificial Intelligence, ICTAI; 2020.	Not about COVID-19 vaccines
37. Lin YJ, Chou WJ, Chang YP, Yen CF. Denial of Justification for Vaccination: Its Multiple Related Variables and Impacts on Intention to Get Vaccinated against COVID-19. *Vaccines (Basel)*. 2021;**9**(8):822. Published 2021 Jul 25. doi:10.3390/vaccines9080822	Not about social media or misinformation
38. Liu S, Liu J. Public attitudes toward COVID-19 vaccines on English-language Twitter: A sentiment analysis. *Vaccine*. 2021;**39**(39):5499-5505. doi:10.1016/j.vaccine.2021.08.058	Not about misinformation
39. Luo S, Xin M, Wang S, et al. Behavioural intention of receiving COVID-19 vaccination, social media exposures and peer discussions in China. *Epidemiol Infect*. 2021;**149**:e158. Published 2021 Apr 23. doi:10.1017/S0950268821000947	Not about misinformation
40. Marcec R, Likic R. Using Twitter for sentiment analysis towards AstraZeneca/Oxford, Pfizer/BioNTech and Moderna COVID-19 vaccines *Postgraduate Medical Journal*. Published Online First: 09 August 2021. doi: 10.1136/postgradmedj-2021-140685	Not about misinformation
41. Marwah HK, Carlson K, Rosseau NA, Chretien KC, Kind T, Jackson HT. Videos, Views, and Vaccines: Evaluating the Quality of COVID-19 Communications on YouTube [published online ahead of print, 2021 Aug 31]. *Disaster Med Public Health Prep*. 2021;1-7. doi:10.1017/dmp.2021.284	Too early in the COVID-19 vaccine development.<br><br>Study from January through June 2020
42. Melki J, Tamim H, Hadid D, Makki M, El Amine J, Hitti E. Mitigating infodemics: The relationship between news exposure and trust and belief in COVID-19 fake news and social media spreading. *PLoS ONE* 2021; 16(6): e0252830. https://doi.org/10.1371/journal.pone.0252830	Too early in the COVID-19 vaccine development.<br><br>Study from March 27 and April 23, 2020.

43. Michel-Kabamba N, Ngatu NR, Leon-Kabamba N, et al. Occupational COVID-19 Prevention among Congolese Healthcare Workers: Knowledge, Practices, PPE Compliance, and Safety Imperatives. *Trop Med Infect Dis*. 2020;**6**(1):6. Published 2020 Dec 30. doi:10.3390/tropicalmed6010006.	Not about COVID-19 vaccines or misinformation.
44. Mo PK, Luo S, Wang S, et al. Intention to Receive the COVID-19 Vaccination in China: Application of the Diffusion of Innovations Theory and the Moderating Role of Openness to Experience. *Vaccines (Basel)*. 2021;**9**(2):129. Published 2021 Feb 5. doi:10.3390/vaccines9020129.	Not about misinformation.
45. Montalti M, Rallo F, Guaraldi F, et al. Would Parents Get Their Children Vaccinated Against SARS-CoV-2? Rate and Predictors of Vaccine Hesitancy According to a Survey over 5000 Families from Bologna, Italy. *Vaccines (Basel)*. 2021;**9**(4):366. Published 2021 Apr 10. doi:10.3390/vaccines9040366	Not about misinformation
46. Moore AC, Freeman-Daily J, Norris K, Bunn B, Baranski J, Chin C, et al. Collaborating to share evidence-basedCOVID-19 information across lung cancer patientadvocacy groups. Clinical Cancer Research Conference: AACR Virtual Meeting: COVID. 2020;26(18 SUPPL).	Not about COVID-19 vaccine or misinformation.
47. Motahari-Nezhad H, Shekofteh M, Andalib-Kondori M. Social media as a platform for information and support for coronavirus: analysis of COVID-19 Facebook groups. Global Knowledge, Memory and Communication. 2021.	Not about misinformation
48. Nazar S, Pieters T. *Plandemic* Revisited: A Product of Planned Disinformation Amplifying the COVID-19 "infodemic". *Front Public Health*. 2021;**9**:649930. Published 2021 Jul 14. doi:10.3389/fpubh.2021.649930	Too early in the COVID-19 vaccine development.<br><br>Study from March 3rd to June 10th, 2020.
49. Nazlı ŞB, Yığman F, Sevindik M, Deniz Özturan D. Psychological factors affecting COVID-19 vaccine hesitancy [published online ahead of print, 2021 May 14]. *Ir J Med Sci*. 2021;1-10. doi:10.1007/s11845-021-02640-0	Not about misinformation
50. Nomura S, Eguchi A, Yoneoka D, et al. Reasons for being unsure or unwilling regarding intention to take COVID-19 vaccine among Japanese people: A large cross-sectional national survey. *Lancet Reg Health West Pac*. 2021;**14**:100223. doi:10.1016/j.lanwpc.2021.100223	Not about misinformation
51. Paul N, Gokhale SS, editors. Analysis and Classification of Vaccine Dialogue in the Coronavirus Era. Proceedings - 2020 IEEE International Conference on Big Data, Big Data 2020; 2020.	Not an empirical study
52. Piltch-Loeb R, Savoia E, Goldberg B, Hughes B, Verhey T, Kayyem J, et al. (2021) Examining the effect of information channel on COVID-19 vaccine acceptance. *PLoS ONE* 16(5): e0251095. https://doi.org/10.1371/journal.pone.0251095	Not about misinformation.
53. Platonov K, Svetlov K. Conspiracy Theories Dissemination on SNS Vkontakte: COVID-19 Case. *Commun Comput Inf Sci* 2020; 322-35.	Too early in the COVID-19 vaccine development. Study from March 30 and May 12, 2020.
54. Qamar M, Irfan O, Dhillon R A, et al. Acceptance of COVID-19 Vaccine in Pakistan: A Nationwide Cross-Sectional Study. *Cureus* 2021;**13**(7): e16603. doi:10.7759/cureus.16603.	Not about misinformation
55. Reno C, Maietti E, Di Valerio Z, Montalti M, Fantini MP, Gori D. Vaccine Hesitancy towards COVID-19 Vaccination: Investigating the Role of Information Sources through a Mediation Analysis. *Infect Dis Rep*. 2021;**13**(3):712-723. Published 2021 Aug 12. doi:10.3390/idr13030066	Not about misinformation
56. Rovetta A. The Impact of COVID-19 on Conspiracy Hypotheses and Risk Perception in Italy: Infodemiological Survey Study Using Google Trends. *JMIR Infodemiology*. 2021;**1**(1):e29929. Published 2021 Aug 6. doi:10.2196/29929	Not about social media.<br><br>Removed during data extraction.
57. Ruiz JB, Bell RA. Predictors of intention to vaccinate against COVID-19: Results of a nationwide survey. *Vaccine*. 2021;**39**(7):1080-1086. doi:10.1016/j.vaccine.2021.01.010	Removed during data extraction.<br><br>Study from June 2020.
58. Sajjadi NB, Nowlin W, Nowlin R, et al. United States internet searches for "infertility" following COVID-19 vaccine misinformation. *Journal of Osteopathic Medicine* 2021;**121**(6):583-587. DOI: 10.1515/jom-2021-0059. PMID: 33838086.	Not about social media
59. Sattar NS, Arifuzzaman S. Covid-19 vaccination awareness and aftermath: Public sentiment analysis on twitter data and vaccinated population prediction in the usa. *Appl Sci* (Switzerland). 2021;**11**(13). https://doi.org/10.3390/app11136128	Not about social media
60. Scannell D, Desens L, Guadagno M, et al. COVID-19 Vaccine Discourse on Twitter: A Content Analysis of Persuasion	Not about COVID-19 vaccine.

Techniques, Sentiment and Mis/Disinformation. *J Health Commun* 2021; **26**:7, 443-459, DOI: 10.1080/10810730.2021.1955050	
61. Sear RF, Velasquez N, Leahy R, et al. Quantifying COVID-19 Content in the Online Health Opinion War Using Machine Learning. *IEEE Access*. 2020;**8**:91886-91893. Published 2020 May 11. doi:10.1109/ACCESS.2020.2993967	Not about COVID-19 vaccine.
62. Shahrezaye M, Meckel M, Steinacker L, Suter V. COVID-19's (Mis)Information Ecosystem on Twitter: How Partisanship Boosts the Spread of Conspiracy Narratives on German Speaking Twitter. Advances in Intelligent Systems and Computing 2021. p. 1060-73.	Not about COVID-19 vaccine.
63. Soveri A, Karlsson LC, Antfolk J, Lindfelt M, Lewandowsky S. Unwillingness to engage in behaviors that protect against COVID-19: the role of conspiracy beliefs, trust, and endorsement of complementary and alternative medicine. *BMC Public Health*. 2021;**21**(1):684. Published 2021 Apr 8. doi:10.1186/s12889-021-10643-w	Too early in the COVID-19 vaccine development**.**<br><br>Study from 3rd and 17th of April 2020
64. Syed Alwi SAR, Rafidah E, Zurraini A, Juslina O, Brohi IB, Lukas S. A survey on COVID-19 vaccine acceptance and concern among Malaysians. *BMC Public Health*. 2021;**21**(1):1129. Published 2021 Jun 12. doi:10.1186/s12889-021-11071-6	Not about misinformation
65. Talukdar D, Stojkovski K, Suarez DB, Gupta MM. Role of Information Technology in COVID-19 Vaccination Drive: An Analysis of the COVID-19 Global Beliefs, Behaviors, and Norms Survey. *Cureus*. 2021;**13**(6):e15922. Published 2021 Jun 25. doi:10.7759/cureus.15922	Not about social media or misinformation.
66. Tan HW, Lee CS, Goh DHL, Zheng H, Theng YL. Analyzing COVID-19 Vaccine Tweets for Tonal Shift. *23rd International Conference on Human-Computer Interaction, HCII 2021 ; 1421:615-623, 2021.*	Not about misinformation.
67. Temsah MH, Barry M, Aljamaan F, et al. SARS-CoV-2 B.1.1.7 UK Variant of Concern Lineage-Related Perceptions, COVID-19 Vaccine Acceptance and Travel Worry Among Healthcare Workers. *Front Public Health*. 2021;**9**:686958. Published 2021 May 26. doi:10.3389/fpubh.2021.686958	Not about misinformation.
68. To QG, To KG, Huynh VN, et al. Applying Machine Learning to Identify Anti-Vaccination Tweets during the COVID-19 Pandemic. *Int J Environ Res Public Health*. 2021;**18**(8):4069. Published 2021 Apr 12. doi:10.3390/ijerph18084069	Not about misinformation.
69. Viswanath K, Hiremath RN, Basra SS, Chourey N. Coronavirus disease 2019 vaccination drive: The perceptions and acceptance of vaccination among health care workers. Asian Journal of Pharmaceutical and Clinical Research. 2021;**14**(6):177-81.	Not about social media or misinformation.
70. Vojtek I, Palsenbarg V, Smyser J. 65. Vaccine Confidence, COVID19, and the Influence of Peer Networks. *Open Forum Infect Dis*. 2020;**7**(Suppl 1):S164. Published 2020 Dec 31. doi:10.1093/ofid/ofaa439.375	Not about misinformation.
71. Wang Z, She R, Chen X, et al. Parental acceptability of COVID-19 vaccination for children under the age of 18 years among Chinese doctors and nurses: a cross-sectional online survey. *Hum Vaccin Immunother*. 2021;**17**(10):3322-3332. doi:10.1080/21645515.2021.1917232	About a future COVID-19 vaccine.<br><br>Not about misinformation.
72. Woko C, Siegel L, Hornik R. An Investigation of Low COVID-19 Vaccination Intentions among Black Americans: The Role of Behavioral Beliefs and Trust in COVID-19 Information Sources. *J Health Commun*. 2020;**25**(10):819-826. doi:10.1080/10810730.2020.1864521	Not about misinformation
73. Yu Y, Lau JTF, Lau MMC, Wong MCS, Chan PKS. Understanding the prevalence and associated factors of behavioral intention of COVID-19 vaccination under specific scenarios combining effectiveness, safety, and cost in the Hong Kong Chinese general population. *Int J Health Policy Manag* 2021. doi:10.34172/ijhpm.2021.02	Not about misinformation.
74. Zeballos Rivas DR, Lopez Jaldin ML, Nina Canaviri B, Portugal Escalante LF, Alanes Fernández AMC, Aguilar Ticona JP. Social media exposure, risk perception, preventive behaviors and attitudes during the COVID-19 epidemic in La Paz, Bolivia: A cross sectional study. *PLoS One* 2021;**16**(1):e0245859.	Too early in the COVID-19 vaccine development.<br><br>Study from April 29th to May 9th 2020.
75. Zhang S, Pian W, Ma F, Ni Z, Liu Y. Characterizing the COVID-19 Infodemic on Chinese Social Media: Exploratory Study. *JMIR Public Health Surveill* 2021;**7**(2):e26090.	Not about COVID-19 vaccine.
